# Stillbirth risk prediction using machine learning for a large cohort of births from Western Australia, 1980–2015

**DOI:** 10.1038/s41598-020-62210-9

**Published:** 2020-03-24

**Authors:** Eva Malacova, Sawitchaya Tippaya, Helen D. Bailey, Kevin Chai, Brad M. Farrant, Amanuel T. Gebremedhin, Helen Leonard, Michael L. Marinovich, Natasha Nassar, Aloke Phatak, Camille Raynes-Greenow, Annette K. Regan, Antonia W. Shand, Carrington C. J. Shepherd, Ravisha Srinivasjois, Gizachew A. Tessema, Gavin Pereira

**Affiliations:** 1 School of Public Health, Curtin University, Perth, WA Australia; 2 QIMR Berghofer Medical Research Institute, Brisbane, QLD Australia; 3 Curtin Institute for Computation, Curtin University, Perth, WA Australia; 4 Telethon Kids Institute, The University of Western Australia, Perth, WA Australia; 5 Child Population and Translational Health Research, The Children’s Hospital at Westmead Clinical School, The University of Sydney, Sydney, NSW Australia; 6 University of Sydney, Sydney School of Public Health, Sydney, NSW Australia; 7 School of Public Health, Texas A&M University, Texas, USA; 8 Department of Maternal Fetal Medicine, Royal Hospital for Women, Randwick, NSW Australia; 9 Ngangk Yira: Murdoch University Research Centre for Aboriginal Health and Social Equity, Perth, WA Australia; 10 Department of Neonatology, Ramsay Health Care, Joondalup Health Campus, Joondalup, WA Australia; 11 Centre for Fertility and Health (CeFH), Norwegian Institute of Public Health, Oslo, Norway; 12 Faculty of Health and Medical Sciences, School of Population and Public Health, Perth, WA Australia; 13 Centre for Transforming Maintenance through Data Science, Curtin University, Perth, WA Australia

**Keywords:** Health services, Population screening

## Abstract

Quantification of stillbirth risk has potential to support clinical decision-making. Studies that have attempted to quantify stillbirth risk have been hampered by small event rates, a limited range of predictors that typically exclude obstetric history, lack of validation, and restriction to a single classifier (logistic regression). Consequently, predictive performance remains low, and risk quantification has not been adopted into antenatal practice. The study population consisted of all births to women in Western Australia from 1980 to 2015, excluding terminations. After all exclusions there were 947,025 livebirths and 5,788 stillbirths. Predictive models for stillbirth were developed using multiple machine learning classifiers: regularised logistic regression, decision trees based on classification and regression trees, random forest, extreme gradient boosting (XGBoost), and a multilayer perceptron neural network. We applied 10-fold cross-validation using independent data not used to develop the models. Predictors included maternal socio-demographic characteristics, chronic medical conditions, obstetric complications and family history in both the current and previous pregnancy. In this cohort, 66% of stillbirths were observed for multiparous women. The best performing classifier (XGBoost) predicted 45% (95% CI: 43%, 46%) of stillbirths for all women and 45% (95% CI: 43%, 47%) of stillbirths after the inclusion of previous pregnancy history. Almost half of stillbirths could be potentially identified antenatally based on a combination of current pregnancy complications, congenital anomalies, maternal characteristics, and medical history. Greatest sensitivity is achieved with addition of current pregnancy complications. Ensemble classifiers offered marginal improvement for prediction compared to logistic regression.

## Introduction

Stillbirth is a devastating outcome for families and society, and accounts for two thirds of perinatal mortality^1–3^. Advances in maternity care since the 1940s have successfully reduced the stillbirth rate by up to 80% in developed countries^4^. However, since then, the stillbirth rate in Australia has remained relatively stable, with a small increase over the 20-year period between 1993 and 2012 (6.4 to 7.2 per 1000 births)^2^, followed by no change between 2013 and 2014 at 7.1 per 1000 births^1^.

Identification of pregnancies at elevated risk of stillbirth is challenging because studies are often hampered by small sample sizes (typically less than approximately 500 stillbirths), a limited number of maternal predictors, and exclusion of multiple gestations, a known high-risk group of potentially preventable stillbirths^5–9^. In addition, although a history of obstetric complications is likely to be associated with stillbirth in multiparous women^10^, obstetric history is rarely considered in stillbirth prediction models. When specificity is fixed at 90%, the stillbirth detection rate (sensitivity) has been reported to vary from 25% using maternal characteristics and chronic conditions alone^8^ to 55% when using clinical tests at the end of second trimester^6^. For these predictive models, the area under the receiver operating curve (AUC) has been shown to range between 66% and 75%, reflecting moderate discrimination. Only one study, conducted in Nigeria, showed greater discrimination with an AUC of 82%^7^, but reported no sensitivity and specificity measures. Moreover, these results were derived in a lower middle income country, and comparability with other studies remains limited. To date, most studies have produced poor performing models for stillbirth prediction, models that have been validated with the same records used for model development, and models that use non-routinely collected risk factors, and therefore have little value for decision support in the antenatal care period.

Routinely collected perinatal records offer great potential to improve identification of pregnancies at elevated risk of stillbirth. Longitudinal record linkage enables the ascertainment of family and obstetric histories. Linkage with hospitalisations widens the predictor set to health conditions that occur between pregnancies and linkage to registries of developmental anomalies extends the predictor set further to birth defects diagnosed up to six years after birth. In Western Australia (WA), these records are comprehensive, with nearly complete population coverage for more than three decades, which allows family obstetric histories to be established for two to three generations.

Advances in computation and the increased availability of open-source software have created the opportunity to improve stillbirth prediction using machine learning. Logistic regression is the most common approach to classification in the stillbirth prediction literature, but this approach has not been compared with alternatives. Side-by-side application of other complementary machine learning approaches to classification will elucidate the value of alternative machine learning classifiers, if any. Certain machine learning classifiers have greater potential to exploit complex non-linear interactions between risk factors that might translate to substantial improvements in stillbirth prediction.

Some stillbirths occur because expectant management is the default for antenatal care and pregnancies at elevated risk of stillbirth go undetected. Effective risk quantification will alert antenatal care providers to pregnancies at-risk that can benefit from intervention. Existing interventions include aspirin for the prevention of pre-eclampsia^11–14^, treatment for gestational diabetes or induction of labour for near or post-term pregnancies^15^. The most substantial gains to perinatal health will be achieved by improving risk detection among asymptomatic pregnant women, or better still, in women prior to pregnancy, and this has not yet been achieved. The primary aim of this study was to quantify and validate the predictive accuracy of a comprehensive range of routinely collected risk factors for predicting stillbirth, including maternal characteristics and chronic medical conditions in current and previous pregnancies, obstetric complications and family history of stillbirth. The secondary aim of this study was to compare the ability of a complementary set of machine learning classifiers to predict stillbirth.

## Methods

### Data sources

The study data and linkage keys were sourced from core and other population health data sets held by the Data Linkage Branch (DLB) of the WA Department of Health, which were combined by probabilistic linkage using common identifiers^16,17^. Birth records for the period from January 1, 1980 to December 31, 2015 were ascertained from the WA Midwives Notifications System, a statutory data collection for all registered livebirths and stillbirths ≥20 weeks of gestation or 400 grams birthweight in WA, and complemented with records from the WA Births Registry. We merged these records to the WA Hospital Morbidity System, WA Register of Developmental Anomalies, and WA Family Connections. Records of terminations of pregnancy were excluded from the study. Extracted records for the cohort included information on maternal demographics, chronic medical conditions, obstetric complications, and infants’ characteristics.

### Outcomes and risk factors

Stillbirth was defined as fetal death from at least 20 weeks of gestation.

Predictors included maternal socio-demographic characteristics, chronic medical conditions, obstetric complications and family history in both the current and previous pregnancy (Supplementary Table 1). Maternal socio-demographic characteristics included age (<20, 20–24, 25–29, 30–34, 35–39, or ≥40 years), ethnicity (Caucasian, Indigenous, or other), urbanicity (rural or urban), area-level socioeconomic status, and parity (0, 1, 2, or ≥3). The Index of Relative Socioeconomic Disadvantage (IRSD) at a Statistical Local Area based on maternal residence at the time of birth was used as a proxy for socioeconomic status and ascertained from the Australian Bureau of Statistics^18^. The IRSD values were divided into quintiles for the state, with the first category assigned as the most disadvantaged. Chronic medical conditions included pre-existing diabetes mellitus, essential hypertension, asthma, obesity, circulatory system disease and any maternal cancer registration prior to or during pregnancy.

Obstetric complications included placenta praevia, unspecified antepartum haemorrhage, pre-labour rupture of membranes, preeclampsia, threatened miscarriage, threatened preterm birth, urinary tract infection, gestational hypertension, and gestational diabetes.

Other characteristics of current pregnancy included: plurality, year of birth, presence of congenital anomaly, and small-for-gestational-age (SGA) birth as a proxy for fetal growth restriction. Congenital anomaly was defined as any birth defect registered in the WA Register of Developmental Anomalies which includes all diagnoses up to six years of age and is comprehensively ascertained from multiple sources in WA. We calculated SGA as the lowest 10^th^ percentile of birthweight by sex and gestation within calendar intervals of five years. For women with two or more birth records during the study period, we derived a history of obstetric complications, indicating whether complications occurred in the last birth, not in the last but in an earlier birth, no complication history, or unknown. This also included previous stillbirth, previous caesarean section, previous gestational diabetes, and previous gestational age (<28, 28–31, 32–36, or 37 or more weeks). Other characteristics of past pregnancies included previous miscarriage (yes, no, or unknown), and previous congenital anomaly (yes, no, or unknown). For births to mothers and fathers who were born during the study period and whose birth details were included in our cohort, we derived parental (maternal or paternal) SGA, parental preterm birth, and parental congenital anomaly. For these parents, we also derived chronic conditions and a history of obstetric complications of their mothers.

### Statistical analysis

We developed three different sets of prediction models based on the total availability of data for each group of relevant predictors (Supplementary Table 1). For these models, we allowed for the inclusion of predictors ascertained after birth that are proxies for measurements taken during pregnancy. The first model (Model A, N = 953,909, stillbirth = 6,836) produced predictions based on maternal socio-demographic characteristics, chronic maternal medical conditions, obstetric complications in current pregnancy, and other characteristics of current pregnancy. The second model (Model B, N = 465,327, stillbirth = 3,110) used predictors from Model A plus obstetric complications in past pregnancies, and other characteristics of past pregnancies. The third model (Model C, N = 136,527, stillbirth = 915) contained all predictors from Model A plus family history. Next we developed another three different sets of prediction models based on the temporal availability of data for each group of relevant predictors. For these models, we did not use predictors unless they were ascertained at that time point in pregnancy or earlier. For these models we also did not use predictors ascertained after birth such as: SGA, which is a proxy for fetal growth measurements taken during pregnancy; and congenital anomalies, which are present *in utero* but can be diagnosed up to 6 years after birth. The fourth model (Model D, N = 952,813, stillbirth = 5,788) contained only predictors collected at the booking appointment (Supplementary Table 1). The fifth model (Model E, N = 464,778, stillbirth = 2,587) contained predictors from model D plus previous pregnancy history. The final model (Model F, N = 464,778, stillbirth = 2,587) contained predictors from model E plus obstetric complications in current pregnancy, and other characteristics of current pregnancy known before delivery.

We used five different machine learning algorithms to perform binary classification as stillbirth versus livebirth. The classifiers considered in this study included: (i) regularised logistic regression, (ii) decision trees based on classification and regression trees (CART), (iii) random forest, (iv) extreme gradient boosting (XGBoost), and (v) a multilayer perceptron (MLP) neural network^19^.

First, we applied regularised logistic regression, which used an L1 penalty to select a subset of predictor variables from a larger ensemble. Estimation proceeds by penalised maximum likelihood, in which the addition of the L1 penalty results in variable selection and shrinkage of the values of the coefficients in the linear predictor^20^. The result combines the interpretability of conventional logistic regression with a model that avoids overfitting.

Second, we applied CART, a tree algorithm. The CART approach for decision trees with the S*cikit-learn* package^19^ undertakes classification through successive splits of the sample into two or more homogeneous sub-samples based on the most significant differentiator at each node using an appropriate impurity criterion (Gini impurity and information gain using entropy). Decision trees are a well-established approach for classification that can produce interpretable results and a hierarchy of *feature* (predictor) importance.

Third, the random forests approach addresses the problem of overfitting with decision trees^21^. A random forest consists of an ensemble of decision trees, in which each tree is trained on random subsets of training samples and predictors. Predictions from these trees are then combined to reduce prediction variance and hence yield more accurate prediction than single decision trees.

Next, we applied boosting, which can improve the predictive power when dealing with high-dimensional data due to a large number of predictors^22^. Boosting combines multiple *weak learners* (i.e. weak prediction rules) to build strong learners. However, the drawback of classical boosting techniques, such as conventional gradient tree boosting, is increased computational time for training the model because the sequential nature of boosting prevents the implementation of more efficient parallelised algorithms^23^. To address this, we applied a more computationally efficient approach developed based on the gradient boosting implementation, XGBoost, which also has an inherent regularisation function that reduces overfitting^24^.

Finally, we applied MLP, which is an artificial neural network that can capture non-linear associations with predictors and statistical interactions between predictors^23,25^. MLPs consist of input layers, one or more hidden layers, and output layers. Within each layer are nodes that connect in one direction, starting from the input layer, passing through the hidden layers, and ending at the output layer. The nodes of the input layer are the predictor variables. Other layers contain nodes that are non-linear functions of weighted combinations of nodes in the previous layer. The output layer produces the stillbirth predictions.

The robustness of predictions was assessed by validation using data not used to develop the models. The dataset was randomly divided into 10 *folds* (i.e. subsets). We employed the *stratified K-folds* approach to generate folds of data that preserved the percentage of stillbirths^26^. The procedure resulted in 10 different training and testing samples. The validation was therefore conducted 10 times, and consequently 10 different classifiers were built for each model and we measured the overall performance of each model by averaging the value of each evaluation metric. Because of the relatively large sample in this study, we could employ cross-validation, which ensured that the data used to develop the model were independent of the data used to evaluate model performance. Hyper-parameter tuning was carried out using an exhaustive exploration of parameter configurations (Supplementary Table 3). The parameter tuning in this study aimed to maximise the positive predictive value (PPV) and sensitivity (true positive rate; TPR) with 10-fold cross-validation (*GridSearchCV*, *Scikit-learn* model selection package)^27^.

The performance of each model was defined as the ability to discriminate between stillbirths and livebirths and was evaluated using a range of metrics from the 10-fold cross-validation. These included the area under the curve (AUC), sensitivity, PPV, negative predictive value (NPV), positive likelihood ratio (LR+), negative likelihood ratio (LR-), and overall accuracy. Overall accuracy was defined as the proportion of all correctly classified subjects. The AUC and accuracy were interpreted as *excellent* (0.9, 1), *good* (0.8, 0.9), *fair* (0.7, 0.8), poor (0.6, 0.7), and *test not useful* (0.5, 0.6). For comparison with past studies, we produced results when the false positive rate (FPR) was held constant at 5% (95% specificity) and 10% (90% specificity)^5,6^. Because the number of livebirths greatly exceeded the number of stillbirths, overall accuracy could be maximised by classification of all births as liveborn. However, this approach has little value for classification of stillbirths. Consequently, we focussed description of results on sensitivity and PPV.

We ascertained sensitivity of our results by repeating analyses for the base predictive model (Model A) after restricting to singletons (A1), restricting to births without congenital malformations (A2), restricting to recent births from 2000 (A3), and restricting to births from 28 weeks gestation (A4) (Supplementary Table 5).

All analyses were conducted with *Anaconda* version 4.5.11 and *Scikit-learn*^19^ version 0.19.1.

The study was approved by the Human Research Ethics Committee of the Department of Health WA and Curtin University. All research activities were conducted in accordance with corresponding guidelines and regulations. Informed consent was waived by these ethics committees on the basis that: (i) the only record linking the participant to the research would be the consent document; (ii) this study uses existing registry data and presents minimal risk of harm to participants; (iii) further direct contact with participants to obtain consent has potential to introduce emotional distress; (iv) the research has potential to lead to public benefit; (v) it is impracticable to obtain informed consent for cohort of this size (N = 947,073 births); and (vi) it is infeasible to obtain informed consent for participants early in the cohort period (1980–2015).

## Results

The final total study population consisted of 947,025 livebirths and 7,788 stillbirths (Supplementary Fig. 1). Stillbirth decreased over time (Supplementary Fig. 2), notably for the Caucasian population (Supplementary Fig. 3). The distributions for the characteristics of the study population (Supplementary Table 1) remained relatively consistent over time (Supplementary Table 4).

For all models, the AUCs varied from 0.59 (95% CI: 0.58, 0.60) to 0.84 (95% CI: 0.83, 0.85), which indicated that discrimination between livebirths and stillbirths was highly dependent on selection of the predictors (Table 1). For all models, the ensemble classifiers (XGBoost and MLP) marginally outperformed the other classifiers in terms of sensitivity and PPV. For Model A, the best performing algorithm (XGBoost) resulted in a sensitivity of 44.6% (95% CI: 43.2%, 46.1%) and PPV of 5.18% (95% CI: 5.02%, 5.33%) at 5% FPR based on predictors including maternal socio-demographic characteristics, chronic maternal medical conditions, obstetric complications in current pregnancy, and other characteristics of current pregnancy. When previous pregnancy history was added (Model B), the best performing algorithm (XGBoost) displayed a slightly improved sensitivity of 45.3% (95% CI: 43.4%, 47.1%) and a PPV of 4.81% (95% CI: 4.63%, 4.99%) at 5% FPR. For the model that included family history (Model C), the best performing algorithm (XGBoost) only achieved a sensitivity of 37.6% (95% CI: 33.6%, 41.6%) and a PPV of 3.86% (95% CI: 3.44%, 4.28%) at 5% FPR.

**Table 1:** Performance of models for predicting stillbirth using different classification algorithms and 10-fold cross validation.

Classifiers	Model	AUC	5% FPR						10% FPR					
			+LR	−LR	Sensitivity	PPV	NPV	CorrectlyClassified	+LR	−LR	Sensitivity	PPV	NPV	Correctly Classified
Logistic Regression	A	0.830	8.10	0.63	40.5	4.72	99.62	94.67	5.52	0.50	55.2	3.26	99.7	89.79
	B	0.834	8.07	0.63	40.5	4.32	99.65	94.68	5.57	0.49	55.7	3.02	99.73	89.80
	C	0.811	7.59	0.66	37.8	3.89	99.65	94.72	5.14	0.54	51.6	2.67	99.71	89.75
	D	0.602	2.25	0.93	11.2	1.35	99.43	94.49	1.90	0.90	19.0	1.15	99.45	89.57
	E	0.633	3.29	0.88	16.5	1.80	99.51	94.54	2.44	0.84	24.4	1.35	99.53	89.64
	F	0.799	6.02	0.74	30.1	3.26	99.59	94.64	4.65	0.60	46.4	2.53	99.67	89.76
Decision Tree	A	0.819	8.16	0.62	40.7	4.75	99.62	94.67	5.68	0.51	54.1	3.35	99.69	90.24
	B	0.808	8.18	0.63	40.6	4.38	99.65	94.73	5.01	0.51	54.7	2.73	99.72	88.88
	C	0.776	6.98	0.68	35.8	3.59	99.64	94.58	5.19	0.63	42.3	2.69	99.67	91.40
	D	0.589	2.07	0.95	10.2	1.25	99.43	94.54	1.78	0.91	17.7	1.08	99.45	89.60
	E	0.599	3.16	0.89	15.2	1.73	99.50	94.68	2.33	0.86	23.0	1.29	99.52	89.67
	F	0.779	5.94	0.74	30.1	3.22	99.59	94.58	5.71	0.73	31.2	3.09	99.59	94.13
Random Forest	A	0.831	8.12	0.63	40.6	4.73	99.62	94.67	5.55	0.50	55.5	3.28	99.70	89.79
	B	0.836	8.22	0.62	41.1	4.40	99.65	94.71	5.66	0.48	56.4	3.07	99.73	89.85
	C	0.788	7.29	0.67	36.4	3.74	99.64	94.69	4.91	0.57	49.1	2.55	99.70	89.78
	D	0.594	2.09	0.94	10.4	1.26	99.43	94.48	1.75	0.92	17.5	1.06	99.44	89.57
	E	0.633	2.87	0.90	14.4	1.58	99.50	94.54	2.37	0.85	23.7	1.31	99.53	89.64
	F	0.801	5.96	0.74	29.8	3.23	99.59	94.64	4.66	0.59	46.7	2.54	99.67	89.76
XGBoost	A	0.840	8.93	0.58	44.6	5.18	99.65	94.70	5.81	0.47	58.1	3.43	99.72	89.81
	B	0.842	9.03	0.58	45.3	4.81	99.68	94.71	5.86	0.46	58.7	3.18	99.74	89.82
	C	0.804	7.54	0.66	37.6	3.86	99.65	94.69	5.12	0.54	51.2	2.66	99.71	89.81
	D	0.596	2.18	0.94	10.9	1.32	99.43	94.49	1.85	0.91	18.5	1.12	99.45	89.57
	E	0.628	3.31	0.88	16.6	1.82	99.51	94.55	2.47	0.84	24.7	1.36	99.53	89.64
	F	0.805	6.56	0.71	32.8	3.54	99.61	94.66	4.84	0.57	48.4	2.64	99.68	89.76
Multi-layer Perceptron	A	0.836	8.57	0.60	42.8	4.98	99.63	94.69	5.65	0.48	56.5	3.34	99.71	89.80
	B	0.840	8.69	0.60	43.5	4.64	99.67	94.71	5.73	0.48	57.2	3.11	99.73	89.83
	C	0.801	7.38	0.67	36.7	3.78	99.65	94.72	5.12	0.55	50.9	2.65	99.71	89.84
	D	0.595	2.15	0.94	10.8	1.30	99.43	94.49	1.84	0.91	18.4	1.11	99.45	89.56
	E	0.634	3.24	0.88	16.2	1.78	99.51	94.57	2.41	0.84	24.1	1.33	99.53	89.64
	F	0.802	6.43	0.71	32.1	3.47	99.60	94.65	4.81	0.58	48.1	2.62	99.68	89.77

Estimates with 95% confidence intervals are provided in the Supplementary Material, Supplementary Table 6.

Model A – Socio-demographics, chronic conditions, current pregnancy complications and characteristics.

Model B – Predictors from Model A, plus previous pregnancy history.

Model C – Predictors from Model A, plus grandmother’s pregnancy history, parental birth outcomes.

Model D – Predictors known at the booking appointment.

Model E – Predictors from Model D, plus previous pregnancy history.

Model F – Predictors from Model E, plus current pregnancy complications and characteristics.

Abbreviations: AUC – Area under the receiving-operator characteristic curve; +LR – Positive likelihood ratio; -LR – Negative likelihood ratio; FPR – alpha (type I error) = 1-specificity; Sensitivity – detection rate, TPR; TP – True Positives; FP – False Positives; TN – True Negatives; FN – False Negatives; PPV – Positive predictive value = TP/(TP + FP); NPV - Negative predictive value = TN/(FN + TN); CI – Confidence Interval.

The worst performing model included predictors known at the booking appointment (Model D). With the best performing algorithm (XGBoost), these predictors only achieved a sensitivity of 10.9% (95% CI: 10.0%, 11.9%) and a PPV of 1.32% (95% CI: 1.21%, 1.43%) at 5% FPR. After the addition of pregnancy history (Model E) to this model, sensitivity increased to 16.6% (95% CI: 14.5%, 18.7%) and PPV increased to 1.82% (95% CI: 1.60%, 2.04%) at 5% FPR. Finally, after further addition of obstetric complications in current pregnancy, as well as other characteristics of current pregnancy known before delivery, the model sensitivity doubled to 32.8% (95% CI: 29.9%, 35.7%) and PPV increased to 3.54% (95% CI: 3.24%, 3.83%) at 5% FPR. The substantial improvement in model performance in Model B compared to Model F was attributable to the inclusion of congenital anomalies and SGA as predictors.

The predictive performances were not sensitive to the inclusion of births with multiple gestations; births with congenital anomalies; births before 28 weeks of gestation; they were not sensitive to the time period of the cohort (Supplementary Table 5).

## Discussion

For our large population-based cohort of almost one million births and nearly 6,000 stillbirths, the XGBoost algorithm was able to use maternal socio-demographic characteristics, chronic maternal medical conditions, obstetric complications in current pregnancy, and other characteristics of the current pregnancy to predict approximately 45% of all stillbirths (at 5% FPR, and 55% at 10% FPR). There was negligible improvement with the inclusion of previous pregnancy history. The addition of the grandmothers’ obstetric history and parental birth outcomes did not improve prediction beyond use of socio-demographics, chronic conditions and current pregnancy complications. However, the inclusion of these predictors changed the population used in the analysis. The availability of grandmothers’ obstetric history implies that the cohort is descendent from family born in Australia. Therefore, stillbirth prediction did not improve either because these predictors provided no additional value to prediction, or because stillbirth prediction was more difficult for the population for which these predictors were available. Although our models can predict almost half of all stillbirths, their PPV remains relatively low at most 5.2% (at 5% FPR). Unlike sensitivity and specificity, PPVs are limited by low prevalence of the outcome (stillbirth). Nonetheless, we have demonstrated that risk factors collected from routine health registries can correctly identify approximately 45 out of 100 stillbirths (at 5% FPR, and 58 out of 100 stillbirths at 10% FPR) and 5 stillbirths per 100 births with a positive test result (at 5% FPR, and 3 stillbirths per 100 births at 10% FPR).

Our study benefited from the inclusion of all births from complete coverage of registered births and midwives notifications, which has been shown to have a high level of validity with strong agreement with medical records^28^. Maternal characteristics, medical history and current complications were systematically recorded and the contribution of risk factors from obstetric and family history was objectively and comprehensively ascertained through record linkage.

We were able to apply and compare the performance for a range of machine learning classifiers. The side-by-side application of multiple machine learning classifiers was a substantial new contribution to the literature given that all past studies employed logistic regression, and that logistic regression was outperformed by the ensemble methods, notably XGBoost. Sensitivity of XGBoost was 4.8 percentage points greater than sensitivity for logistic regression (Model B, 5% FPR). In our study of 5,788 stillbirths, this FPR equates to an additional 278 stillbirths that would have otherwise been missed using logistic regression. Although the ensemble methods outperformed other classifiers, the degree to which improvement was gained must be kept in context. In particular, the range in sensitivity attributable to predictors and populations (models) was more than five times the range in sensitivity attributable to machine learning classifiers (within models).

### Comparison with other studies

Previous studies have shown that maternal factors alone can predict about 16% of all stillbirths at a 5% FPR, and that the addition of screening tests and biomarkers can improve the prediction rate to one third of all stillbirths at the end of first trimester and to nearly half of all stillbirths at the end of second trimester^5,6,9,10^. These tests, which are used for assessment of stillbirth risk in selected populations at risk for anomalies, appear to be relatively more effective for stillbirths attributable to impaired placentation^5,6,9,10^. The most effective tests were found to be uterine artery pulsatility index (UtA-PI), maternal serum pregnancy-associated plasma protein-A (PAPP-A), ductus venosus pulsatility index for veins (DV-PIV) and fetal biometry^5,6^.

Some of the best results have been reported by Akolekar and colleagues in the UK^5,6^. Their prediction models (<300 stillbirths) identified PAPP-A (at 11–13 weeks), DV-PIV (at 11–13 weeks), UtA-PI (at 11–13 weeks, and 19–24 weeks), and fetal biometry (at 19–24 weeks) as the best predictors of stillbirth (defined from at least 24 weeks of gestation)^10^. At a FPR of 5%, these tests combined with maternal factors detected 33% of stillbirths at the end of first trimester (explaining 0.72 AUC), or 45% of stillbirths at the end of second trimester (explaining 0.75 AUC)^6,10^. A slightly larger study of <500 stillbirths by Trudell and colleagues in the US focussed on prediction of late stillbirths ≥32 weeks of gestation^8^. However, their best performing model included very few maternal characteristics and chronic conditions (maternal age, African racial origin, nulliparity, smoking, hypertension, diabetes and body mass index), which could potentially detect either 55% of stillbirths with low specificity of 67% (33% FPR) or 25% of stillbirths with 92% specificity (8% FPR). Although their model had a moderate AUC 0.66 (95% CI: 0.60, 0.72), its wide confidence interval suggests imprecision. Finally, the prediction model developed by Kyaode and colleagues in the Netherlands (<500 stillbirths) for low-resource settings displayed some promising features, although it may be less applicable for detecting stillbirths in high-income countries^7^, given the underlying causes between high- and low-income countries often differ^29^. Although their extended model, which combined growth rate with fetal presentation, bleeding, maternal comorbidity and maternal characteristics, achieved high calibration and discrimination 0.82 AUC (95% CI: 0.80, 0.83), no sensitivity or specificity measures were reported, thus making comparisons with different models difficult, since AUCs tend to increase with smaller sample sizes. Only one study presented results for which the births used to evaluate model performance did not include the births used to build the predictive model^8^.

Stillbirth prediction will benefit greatly from use of cohorts with a large number of stillbirth events, the development of models that incorporate validation using birth records not used to develop the predictive models, the use of clinical predictors whose ascertainment is not a consequence of prior suspicion of stillbirth, determination of the cause of stillbirth and the application of complementary classifiers for prediction.

### Limitations

A limitation of using perinatal records that spanned more than three decades is that the database changed over time and therefore some predictors became available, more detailed, or mandatory later in the study period. However, sensitivity analyses indicated that results did not differ after restriction to births later in the study period. Although we supplemented predictor ascertainment from perinatal records with hospitalisation records, these can under-ascertain prevalence of risk factors in the wider population, e.g., urinary tract infections during pregnancy. In this study, we enumerated risk factors according to their states – known with the condition, known without the condition, or not known. Although this approach does not fully address the issue of missing risk factors, it reflects real-life situations – at any given point in time, not all risk factors are known.

We were unable to classify stillbirths as antepartum versus intrapartum because this information was not available prior to 2005, and thus we were unable to develop a prediction model specifically for antepartum stillbirths. However, based on 2011–2012 data from two Australian states (Victoria and Queensland), approximately 80% of stillbirths are antepartum^2^.

We also did not have information on the exact timing of onset for the risk factors that were used as predictors of stillbirth in the predictive models. To partially address this issue, we produced additional models that sequentially added predictors as they became available during pregnancy, commencing from predictors known at the booking appointment. Ideally, estimates of stillbirth risk would be updated throughout gestation, and it would be expected that predictive performance and clinical relevance would improve as new risk factors are diagnosed.

The accuracy of the predictors also varied throughout gestation. Retrospective extrapolation using factors known after the birth in antenatal prediction models can lead to either overstated or understated predictive ability. Notably, SGA was used as a proxy for fetal growth restriction, which may not have been known prior to the birth or may have been a less accurate proxy than anthropometric measurements from ultrasounds. Our results indicated that the performance of models was dependent on fetal growth restriction and congenital anomaly, which are well-established strong risk factors for stillbirth.

Finally, predictive models for less prevalent events such as stillbirth necessitates the use of large population health records, but these records do not contain information on the indication of antenatal interventions. We cannot rule out the possibility of confounding by indication, which together with multicollinearity means that effect estimates of individual risk factors are less interpretable. It is possible that highly effective treatment or clinical intervention for significant stillbirth risk factors can erroneously make it appear that these risk factors are protective. Therefore, the results performed well for prediction, but caution should be exercised before drawing inference on causation.

### Clinical significance

Although risk of mortality and morbidity is assessed by clinical care providers using risk factors and morbidities ascertained in the antenatal period, there is currently no quantitative risk assessment system to identify and stratify at-risk pregnancies to provide information for evidence-based clinical decision-making. Our study results are promising for the development of decision support systems to identify pregnancies at elevated risk of stillbirth. Use of the predictors is non-intrusive to mothers because they are already routinely collected in antenatal systems. The predictions are not likely to introduce any harm if used as *decision-support* to complement existing antenatal care. Risk prediction could lead to individualised care that may involve ultrasound surveillance for growth, continuity of care, “high risk” care with obstetricians and midwives, or timed birth (induction of labour or pre-labour caesarean section). Our results motivate quantitative risk stratification, the addition of clinical predictors routinely collected during the antenatal period, and a focus on prognostic models through which risk can be continually updated during pregnancy.

## Conclusions

We demonstrated that almost half of stillbirths could be potentially identified antenatally based on a combination of current pregnancy complications, congenital anomalies, maternal characteristics, and medical history, but that the greatest sensitivity is achieved using current pregnancy complications. Some machine learning classifiers (ensemble methods) offer some improvement for prediction compared to logistic regression. Future improvement in quantitative stillbirth risk stratification will be achieved with the addition of information that is routinely collected during the antenatal period, including both risk factors as well as indications for treatment.

## Supplementary information

Supplementary Materials.
