# Supplementary material for: Stillbirth risk prediction using machine learning for a large cohort of births from Western Australia, 1980–2015
Source: Sci Rep. 2020 Mar 24;10:5354. doi: 10.1038/s41598-020-62210-9 (PMC7093523; doi:10.1038/s41598-020-62210-9)
Supplement: Supplementary file 1 — Supplementary Materials. [file 41598_2020_62210_MOESM1_ESM.docx]

**Stillbirth risk prediction using machine learning for a large cohort of births from Western Australia, 1980 – 2015**

Eva Malacova ^1, 2^

Sawitchaya Tippaya ^1, 3^

Helen Bailey ^4^

Kevin Chai ^3^

Brad M. Farrant ^4^

Amanuel Gebremedhin ^1^

Helen Leonard ^4^

Michael L. Marinovich ^1^

Natasha Nassar ^5^

Aloke Phatak ^3^

Camille Raynes-Greenow ^6^

Annette K. Regan ^1, 7^

Antonia W. Shand ^5,8^

Carrington Shepherd ^4, 9^

Ravisha Srinivasjois ^1, 4, 10^

Gizachew A. Tessema^1^

Gavin Pereira^1, 4, 11^

1. School of Public Health, Curtin University, WA, Australia

2. QIMR Berghofer Medical Research Institute, QLD, Australia

3. Curtin Institute for Computation, Curtin University, WA, Australia

4. Telethon Kids Institute, The University of Western Australia, WA, Australia

5. Child Population and Translational Health Research, The Children’s Hospital at Westmead Clinical School, The University of Sydney, New South Wales, Australia

6. University of Sydney, Sydney School of Public Health, NSW, Australia

7. School of Public Health, Texas A&M University, Texas, USA

8. Department of Maternal Fetal Medicine, Royal Hospital for Women, Randwick, NSW Australia

9. Ngangk Yira: Murdoch University Research Centre for Aboriginal Health and Social Equity, Western Australia, Australia

10. Department of Neonatology, Ramsay Health Care, Joondalup Health Campus, WA, Australia

11. Centre for Fertility and Health (CeFH), Norwegian Institute of Public Health, Oslo, Norway

Correspondence to: Associate Professor Gavin Pereira

School of Public Health

Curtin University

Perth, WA 6102, Australia

[Gavin.f.pereira@curtin.edu.au](mailto:Gavin.f.pereira@curtin.edu.au)

+61 8 9266 3940

# Supplementary Figure 1. Selection of birth records for analysis, Western Australia (WA), 1980-2015.

*Reasons for 5,643 births exclusions:*

- 19 duplicates (14 duplicate entries, 5 duplicate dates of birth for singletons)
- 338 duplicates with different root numbers for singletons but the same date of birth and weight and born to the same mother
- 72 births with <20 weeks gestation
- 5,214 births with missing gestational age

957,620 eligible births in total

958,729 births with no duplicates and gestational age of 20 weeks or more

Exclusions of 1109 terminations

964,372 births

Included 3,060 births from WA Birth Registry

961,312 births from WA Midwives Notification System

*Model C:*

Total births: 136,324

Livebirth: 135,601

Stillbirth: 723

*Model B, E, F:*

Total births: 464,778

Livebirth: 462,191

Stillbirth: 2,587

*Model A, D:*

Total births: 952,813

Livebirth: 947,025

Stillbirth: 5,788

*Model C:*

Excluded 3,334 births containing unknown values*

*Model B, E, F:* Excluded 2,887 births containing unknown values*

*Model A, D:* Excluded 4,807 births containing unknown values*

* Births containing unknown values for a subset of predictors were excluded to reduce multicollinearity of predictors. These predictors were: essential hypertension, previous miscarriage, previous threatened preterm birth, previous small-for-gestational- age birth, previous gestational age, parents preterm birth, maternal or paternal grandmothers hypertension, paternal or maternal grandmothers miscarriage, maternal or paternal grandmothers circulatory system disease, maternal or paternal grandmothers cancer registration.

**Supplementary Figure 2. Time series for the stillbirth rate in Western Australia, 1980-2015.**


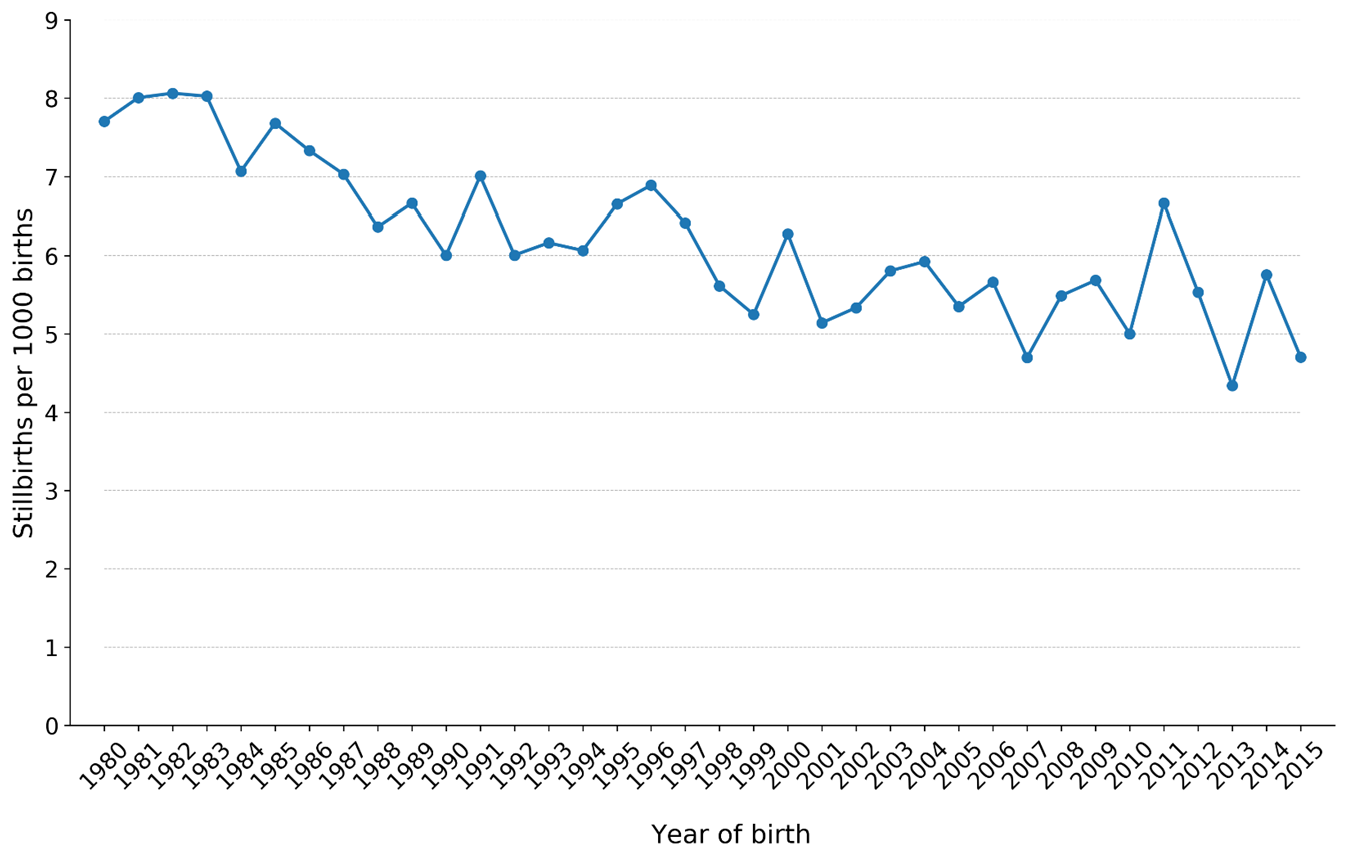


Note: Excludes terminations of pregnancy.

**Supplementary Figure 3. Time series for the stillbirth rate by ethnicity in Western Australia, 1980-2015.**


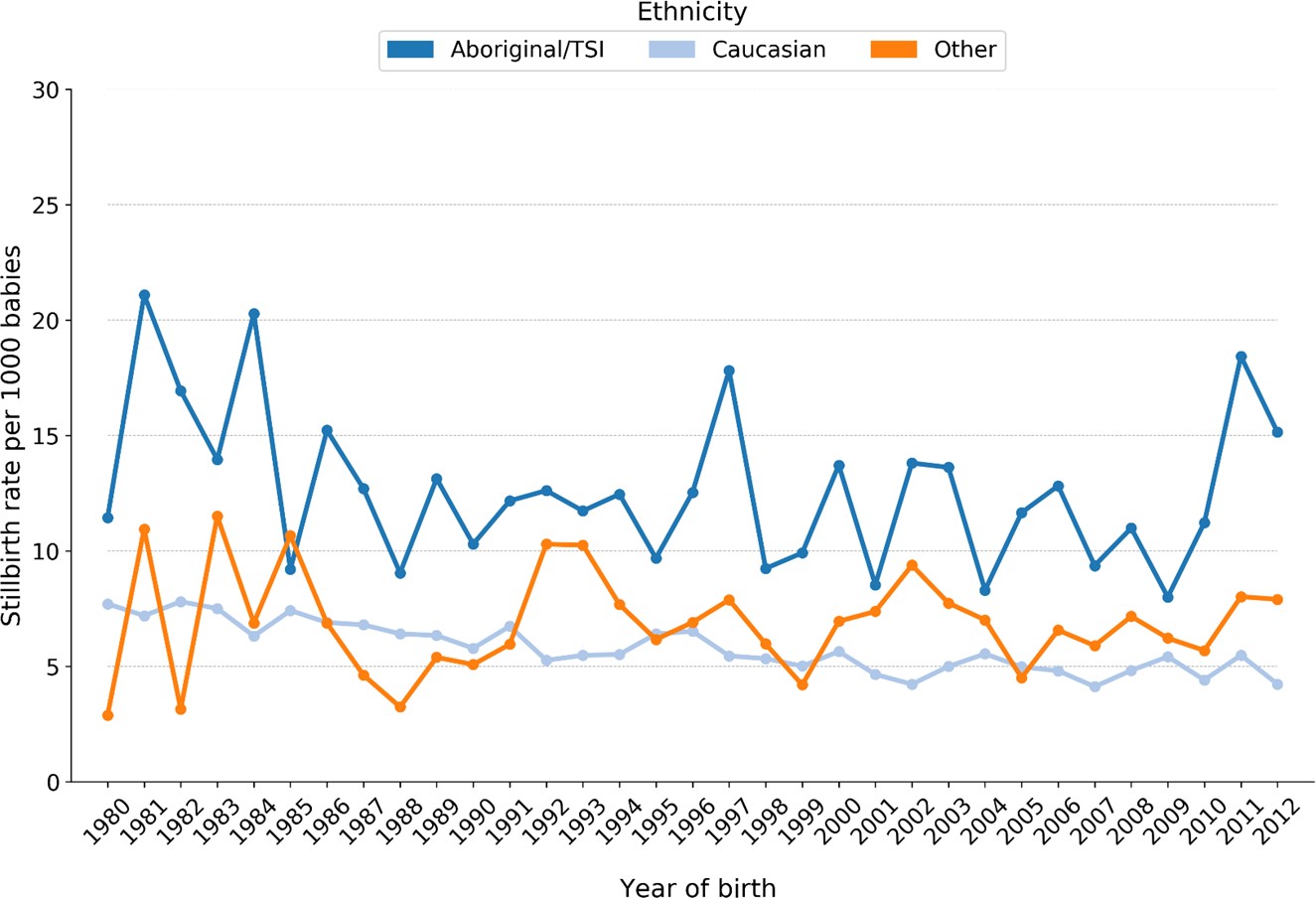


Note: Excludes terminations of pregnancy.

**Supplementary Table 1. Summary of predictors used in this study**

| **Predictor**  **Group** | **Predictors** | **Data**  **Source** | **Description** | **Years of**  **availability** |  |  | **Model** |  |  |  |
| --- | --- | --- | --- | --- | --- | --- | --- | --- | --- | --- |
|  |  |  |  |  | **A** | **B** | **C** | **D** | **E** | **F** |
| Socio- demographics | Maternal age | MNS | 5-year age groups | 1980- 2015 | ✓ | ✓ | ✓ | ✓ | ✓ | ✓ |
|  | Ethnicity | MNS | Caucasian Indigenous Other  Unknown | 1980- 2015  (updated Jan 2013) | ✓ | ✓ | ✓ | ✓ | ✓ | ✓ |
|  | Socioeconomic status | ABS | SEIFA Index of  Socioeconomic Disadvantage (Quintiles) | 1980- 2015 | ✓ | ✓ | ✓ | ✓ | ✓ | ✓ |
|  | Urbanicity | MNS and  Family connection | Urban  Rural Unknown | 1980- 2015 | ✓ | ✓ | ✓ | ✓ | ✓ | ✓ |
|  | Birth year | MNS and Family  connection | 5-year groups | 1980-2015 | ✓ | ✓ | ✓ | ✓ | ✓ | ✓ |
|  | Smoking | MNS and Family connection | Yes No  Unknown | 1997-2015 | ✓ | ✓ | ✓ | ✓ | ✓ | ✓ |
| Chronic conditions | Essential hypertension | HMDS MNS | ICD-9:401, ICD-10: I10 | 1980- 2015 | ✓ | ✓ | ✓ | ✓ | ✓ | ✓ |
|  | Pre-existing diabetes | HMDS MNS | ICD-9:250,ICD-10:E08- E11,E13 | 1980- June  2014 | ✓ | ✓ | ✓ | ✓ | ✓ | ✓ |
|  | Asthma | HMDS  MNS | ICD-9:493,ICD-10:J45 | 1980- 2015 | ✓ | ✓ | ✓ | ✓ | ✓ | ✓ |
|  | Previous miscarriage | HMDS MNS | ICD-9: 634, ICD-10: O03  <20 weeks gestation | 1980- 2015 | ✓ | ✓ | ✓ | ✓ | ✓ | ✓ |
|  | Obesity | HMDS  MNS | ICD-9:278.0-278.03,ICD-  10:E66 | 1980- 2015 | ✓ | ✓ | ✓ | ✓ | ✓ | ✓ |
|  | Circulatory  system disease | HMDS  MNS | ICD-9:390-459,ICD-  10:100-199 | 1980- 2015 | ✓ | ✓ | ✓ | ✓ | ✓ | ✓ |
| Current pregnancy  characteristics | Parity | MNS and  Family connection | Total of previous live births and stillbirths | July 2014-  2015 | ✓ | ✓ | ✓ | ✓ | ✓ | ✓ |
| and |  |  |  |  |  |  |  |  |  |  |
| complications |  |  |  |  |  |  |  |  |  |  |
|  | Gestational | HMD | ICD9:648.8,ICD- | 1980 -2015 | ✓ | ✓ | ✓ |  |  | ✓ |
|  | diabetes | MNS | 10:O24.4 |  |  |  |  |  |  |  |
|  | Gestational | HMDS | ICD9:642.3,ICD-10:O13 | July 2014- | ✓ | ✓ | ✓ |  |  | ✓ |
|  | hypertension | MNS |  | Dec 2015 |  |  |  |  |  |  |
|  | Threatened  miscarriage | HMDS  MNS | ICD-9: 640-640.03, ICD-  10: O20.0 | 1980- 2015 | ✓ | ✓ | ✓ |  |  | ✓ |
|  |  |  | <20 weeks gestation |  |  |  |  |  |  |  |
|  | Preeclampsia | HMDS | ICD-9: 642.4, 642.5, | July 2014- | ✓ | ✓ | ✓ |  |  | ✓ |
|  |  | MNS | 642.7, ICD-10: O14, O11 | 2015 |  |  |  |  |  |  |
|  | Placenta | HMDS | ICD-9: 641.0-641.1, ICD- | 1980- 2015 | ✓ | ✓ | ✓ |  |  | ✓ |
|  | praevia | MNS | 10: O44 |  |  |  |  |  |  |  |
|  | Pre-labour | HMDS | ICD9:658.1,ICD-10:O42 | 1980- 2015 | ✓ | ✓ | ✓ |  |  | ✓ |
|  | rupture of  membranes | MNS |  |  |  |  |  |  |  |  |
|  | Unspecified | HMDS | ICD-9:641.3- | 1980-2015 | ✓ | ✓ | ✓ |  |  | ✓ |
|  | antepartum haemorrhage | MNS | 641.99,640.8-  640.9,ICD10:O46,O20.8- O20.9 |  |  |  |  |  |  |  |
|  | Urinary tract | HMDS | ICD-9: 646.6, ICD-10: | 1980- 2015 | ✓ | ✓ | ✓ |  |  | ✓ |
|  | infection | MNS | O23 |  |  |  |  |  |  |  |
|  | Small-for- gestational age | MNS | <10^th^ centile of birth weights for gestational  week and sex | 1980- 2015 | ✓ | ✓ | ✓ |  |  |  |
|  | Cancer | Cancer | ICD-9: 140-239, ICD-10: | 1980- 2015 | ✓ | ✓ | ✓ |  |  | ✓ |
|  | registration | Registry HMDS | C00-D49  Cancer registration during |  |  |  |  |  |  |  |

|  |  |  | pregnancy |  |  |  |  |  |  |  |
| --- | --- | --- | --- | --- | --- | --- | --- | --- | --- | --- |
|  | Threatened  preterm birth | MNS | Threatened preterm birth  <37 weeks | 1980- 2015 | ✓ | ✓ | ✓ |  |  | ✓ |
|  | Plural | MNS | Singleton  Twin  Multiple gestation > 2 | 1980- 2015 | ✓ | ✓ | ✓ |  |  | ✓ |
|  | Congenital  anomaly | WARDA | Any birth defect | 1980- 2015 | ✓ | ✓ | ✓ |  |  |  |
| Previous  pregnancy history | Previous  stillbirth | Death  Registry | Stillbirth born after 28  weeks gestation | 1980- 2015 |  | ✓ |  |  | ✓ | ✓ |
|  | Previous  gestational diabetes | HMDS  MNS | ICD-9: 648.8, ICD-10:  O24.4 | 1980- 2015 |  | ✓ |  |  | ✓ | ✓ |
|  | Previous  gestational hypertension | HMDS  MNS | ICD-9: 642.3, ICD-10:  O13 | 1980- 2015 |  | ✓ |  |  | ✓ | ✓ |
|  | Previous  threatened miscarriage | HMDS  MNS | ICD-9: 640-640.03, ICD-  10: O20.0 | 1980- 2015 |  | ✓ |  |  | ✓ | ✓ |
|  | Previous  preeclampsia | HMDS  MNS | ICD-9: 642.4, 642.5,  642.7, ICD-10: O14, O11 | 1980- 2015 |  | ✓ |  |  | ✓ | ✓ |
|  | Previous  placenta praevia | HMDS  MNS | ICD-9: 641.0-641.1, ICD-  10: O44 | 1980- 2015 |  | ✓ |  |  | ✓ | ✓ |
|  | Previous  placental abruption | HMDS  MNS | ICD-9: 641.2, ICD-10:  O45 | 1980- 2015 |  | ✓ |  |  | ✓ | ✓ |
|  | Previous pre- labour rupture  of membranes | HMDS MNS | ICD-9: 658.1, ICD-10: O42 | 1980- 2015 |  | ✓ |  |  | ✓ | ✓ |
|  | Previous  unspecified antepartum haemorrhage | HMDS  MNS | ICD-9: 641.3-641.99,  640.8-640.9, ICD-10: O46, O20.8-O20.9 | 1980- 2015 |  | ✓ |  |  | ✓ | ✓ |
|  | Previous urinary tract  infection | HMDS MNS | ICD-9: 646.6, ICD-10: O23 | 1980- 2015 |  | ✓ |  |  | ✓ | ✓ |
|  | Previous  threatened preterm birth | MNS | Previous threatened  preterm birth <37 weeks | 1980-2015 |  | ✓ |  |  | ✓ | ✓ |
|  | Previous  uterine rupture | HMDS  MNS | ICD-9: 665.0-665.1, ICD-  10: O71.0-O71.1 | 1980-2015 |  | ✓ |  |  | ✓ | ✓ |
|  | Previous cancer  registration | Cancer Registry  HMDS | ICD-9: 140-239, ICD-10: C00-D49 | 1980- 2015 |  | ✓ |  |  | ✓ | ✓ |
|  | Previous small-  for-gestational- age | MNS | <10^th^ centile of birth  weight for gestational week and sex | 1980- 2015 |  | ✓ |  |  | ✓ | ✓ |
|  | Previous  congenital anomalies | WARDA | Any birth defect | 1980- 2015 |  | ✓ |  |  | ✓ | ✓ |
|  | Previous  caesarean delivery | MNS  HMDS | Mode of delivery | 1998- 2015 |  | ✓ |  |  | ✓ | ✓ |
|  | Previous gestational age | MNS | <28 weeks  28-31 weeks  32-36 weeks  ≥37 weeks | 1980- 2015 |  | ✓ |  |  | ✓ | ✓ |
| Parental birth outcomes | Parent small- for-gestational- age | MNS | <10^th^ centile of birth weight for gestational week and sex | 1980- 2015 |  |  | ✓ |  |  |  |
|  | Parent preterm  birth | MNS | <37 weeks gestation | 1980- 2015 |  |  | ✓ |  |  |  |
|  | Parent  congenital anomaly | WARDA | Any birth defect | 1980- 2015 |  |  | ✓ |  |  |  |
| Grandmothers pregnancy history | Grandmothers hypertension | HMDS MNS | ICD-9: 401, ICD-10: I10 | 1980- 2015 |  |  | ✓ |  |  |  |

|  | Grandmothers  diabetes | HMDS  MNS | ICD-9:250,ICD-10:E08-  E11,E13 | 1980- 2015 |  |  | ✓ |  |  |  |
| --- | --- | --- | --- | --- | --- | --- | --- | --- | --- | --- |
|  | Grandmothers  miscarriage | HMDS  MNS | ICD-9: 634, ICD-10:O03  <20 weeks gestation | 1980- 2015 |  |  | ✓ |  |  |  |
|  | Grandmothers  asthma | HMDS  MNS | ICD-9:493,ICD-10:J45 | 1980- 2015 |  |  | ✓ |  |  |  |
|  | Grandmothers  circulatory system disease | HMDS  MNS | ICD-9:390-459,ICD-  10:100-199 | 1980- 2015 |  |  | ✓ |  |  |  |
|  | Grandmothers  threatened miscarriage | HMDS  MNS | ICD-9: 640-640.03,  ICD10: O20.0  <20 weeks gestation | 1980- 2015 |  |  | ✓ |  |  |  |
|  | Grandmothers  preeclampsia | HMDS  MNS | ICD-9: 642.4, 642.5,  642.7, ICD-10: O14, O11 | 1980- 2015 |  |  | ✓ |  |  |  |
|  | Grandmothers  placenta praevia | HMDS  MNS | ICD-9: 641.0-641.1, ICD-  10: O44 | 1980- 2015 |  |  | ✓ |  |  |  |
|  | Grandmothers  placental abruption | HMDS  MNS | ICD-9: 641.2, ICD-10:  O45 | 1980- 2015 |  |  | ✓ |  |  |  |
|  | Grandmothers  pre-labour  rupture of membranes | HMDS  MNS | ICD-9: 658.1, ICD-10:  O42 | 1980- 2015 |  |  | ✓ |  |  |  |
|  | Grandmothers  unspecified  antepartum haemorrhage | HMDS  MNS | ICD-9: 641.3-641.99,  640.8-640.9, ICD-10: O46, O20.8-O20.9 | 1980- 2015 |  |  | ✓ |  |  |  |
|  | Grandmothers  gestational diabetes | HMDS  MNS | ICD-9: 648.8, ICD-10:  O24.4 | 1980- 2015 |  |  | ✓ |  |  |  |
|  | Grandmothers urinary tract infection | HMDS MNS | ICD-9: 646.6, ICD-10: O23 | 1980- 2015 |  |  | ✓ |  |  |  |
|  | Grandmothers  cancer registry | Cancer  Registry HMDS | ICD-9: 140-239, ICD-10:  C00-D49 | 1980- 2015 |  |  | ✓ |  |  |  |
|  | Grandmothers history of stillbirth | Death Registry | Stillbirth born after 28 weeks gestation | 1980- 2015 |  |  | ✓ |  |  |  |
|  | Grandmothers  gestational hypertension | HMDS  MNS | ICD-9: 642.3, ICD-10:  O13 | 1980- 2015 |  |  | ✓ |  |  |  |
|  | Grandmothers birth year | MNS and Mother’s morbidity  data | Year of birth for records with grandmothers history | 1980- 2015 |  |  | ✓ |  |  |  |

MNS- Midwives Notification System; HMDS- Hospital Morbidity Data System; ICD codes-International Statistical Classification of Diseases and Related Health Problems; WARDA- Western Australian Registry of Developmental Anomalies

Coding for previous conditions: nulliparous - parity 0; first birth, parity > 0 – no previous records exist because this was the first birth in the study period for a non- nulliparous mother; no history – previous birth records exist and there is no history of the condition; yes, in an earlier birth – the condition was present for an earlier birth; yes, in last birth – the condition was present for the most recent previous birth; unknown – unknown due to missing variables.

Parent – any parent listed on the birth record. Grandmother – Maternal or paternal grandmother

# Supplementary Table 2a. Distribution of maternal characteristics for all births in Western Australia, 1980-2015: Socio-demographics

| **Predictors** | **All births N (%)** | **Livebirth N (%)** | **Stillbirth N (%)** | **Predictors** | **All births N (%)** | **Livebirth N (%)** | **Stillbirth N (%)** |
| --- | --- | --- | --- | --- | --- | --- | --- |
| **SOCIO- DEMOGRAPHICS** | | | |  | | | |
| **Maternal Age** | | | | **Urbanicity** | | | |
| <20 years | 52,336 (5.47) | 51,906 (5.45) | 430 (7.35) | Urban | 634,427 (66.25) | 630,757 (66.27) | 3,670 (62.75) |
| 20-24 years | 181,252 (18.93) | 180,112 (18.92) | 1,140 (19.49) | Rural | 225,969 (23.60) | 224,486 (23.59) | 1,483 (25.35) |
| 25-29 years | 306,642 (32.02) | 304,973 (32.04) | 1,669 (28.53) | Unknown | 97,224 (10.15) | 96,528 (10.14) | 696 (11.90) |
| 30-34 years | 274,699 (28.69) | 273,092 (28.69) | 1,607 (27.47) | **Birth year** | | | |
| 35-39 years | 120,168 (12.55) | 119,386 (12.54) | 782 (13.37) | 1980-1984 | 109,258 (11.41) | 108,409 (11.39) | 849 (14.52) |
| ≥40 years | 22,515 (2.35) | 22,297 (2.34) | 218 (3.73) | 1985-1989 | 121,891 (12.73) | 121,038 (12.72) | 853 (14.58) |
| Unknown | 8 (0.00) | 5 (0.00) | 3 (0.05) | 1990-1994 | 126,968 (13.26) | 126,175 (13.26) | 793 (13.56) |
| **Ethnicity** | | | | 1995-1999 | 127,585 (13.32) | 126,799 (13.32) | 786 (13.44) |
| Caucasian | 797,721 (83.30) | 793,247 (83.34) | 4,474 (76.49) | 2000-2004 | 124,910 (13.04) | 124,198 (13.05) | 712 (12.17) |
| Indigenous | 48,898 (5.11) | 48,287 (5.07) | 611 (10.45) | 2005-2009 | 147,258 (15.38) | 146,466 (15.39) | 792 (13.54) |
| Other | 110,971 (11.59) | 110,221 (11.58) | 750 (12.82) | 2010-2015 | 199,750 (20.86) | 198,686 (20.88) | 1,064 (18.19) |
| Unknown | 30 (0.00) | 16 (0.00) | 14 (0.24) | **Smoking** | | | |
| **Socioeconomic status** | | | | Yes | 83,619 (8.73) | 82,982 (8.72) | 637 (10.89) |
| <20% (low) | 185,911 (19.41) | 184,477 (19.38) | 1,434 (24.52) | No | 447,116 (46.69) | 444,891 (46.74) | 2,225 (38.04) |
| 20-39% | 183,398 (19.15) | 182,234 (19.15) | 1,164 (19.90) | Unknown | 426,885 (44.58) | 423,898 (44.54) | 2,987 (51.07) |
| 40-59% | 182,449 (19.05) | 181,346 (19.05) | 1,103 (18.86) |  |  |  |  |
| 60-79% | 181,018 (18.90) | 180,088 (18.92) | 930 (15.90) |  |  |  |  |
| ≥80% (high) | 178,561 (18.65) | 177,665 (18.67) | 896 (15.32) |  |  |  |  |
| Unknown | 46,283 (4.83) | 45,961 (4.83) | 322 (5.51) |  |  |  |  |

**Supplementary Table 2b. Distribution of maternal characteristics for all births in Western Australia, 1980-2015: Chronic conditions and Current pregnancy characteristics and complications**

| **Predictors** | **All births N (%)** | **Livebirth N (%)** | **Stillbirth N (%)** | **Predictors** | **All births N (%)** | **Livebirth N (%)** | **Stillbirth N (%)** |
| --- | --- | --- | --- | --- | --- | --- | --- |
| **CHRONIC CONDITIONS** | | | | **Circulatory system disease**  Yes | 8,882 (0.93) | 8,801 (0.92) | 81 (1.38) |
| **Essential hypertension** | | | |  |  |  |  |
| Yes | 3,119 (0.33) | 3,080 (0.32) | 39 (0.67) | No | 943,931 (98.57) | 938,224 (98.58) | 5,707 (97.57) |
| No Unknown | 954,474 (99.67)  27 (0.00) | 948,667 (99.67)  24 (0.00) | 5,807 (99.28)  3 (0.05) | Unknown | 4,807 (0.50) | 4,746 (0.50) | 61 (1.04) |
|  |  |  |  | **CURRENT PREGNANCY CHARACTERISTICS AND**  **COMPLICATIONS** | | | |
| **Pre-existing diabetes** | | | | **Parity** | | | |
| Yes | 3,136 (0.33) | 3,066 (0.32) | 70 (1.20) | 0 | 387,156 (40.43) | 384,605 (40.41) | 2,551 (43.61) |
| No | 954,457 (99.67) | 948,681 (99.68) | 5,776 (98.75) | 1 | 322,924 (33.72) | 321,315 (33.76) | 1,609 (27.51) |
| Unknown | 27 (0.00) | 24 (0.00) | 3 (0.05) | 2 | 155,336 (16.22) | 154,445 (16.23) | 891 (15.23) |
| **Asthma** | | | | **>**2 | 92,086 (9.62) | 91,303 (9.59) | 783 (13.39) |
| Yes | 42,158 (4.40) | 41,930 (4.41) | 228 (3.90) | Unknown | 118 (0.01) | 103 (0.01) | 15 (0.26) |
| No | 915,435 (95.59) | 909,817 (95.59) | 5,618 (96.05) | **Gestational diabetes** | | | |
| Unknown | 27 (0.00) | 24 (0.00) | 3 (0.05) | Yes | 42,936 (4.48) | 42,711 (4.49) | 225 (3.85) |
| **Previous miscarriage** | | | | No | 914,679 (95.52) | 909,058 (95.51) | 5,621 (96.10) |
| Yes | 26,486 (2.77) | 26,219 (2.75) | 267 (4.56) | Unknown | 5 (0.00) | 2 (0.00) | 3 (0.05) |
| No | 926,327 (96.73) | 920,806 (96.75) | 5,521 (94.39) |  |  |  |  |
|  |  |  |  | **Gestational** | | | |
| Unknown | 4,807 (0.50) | 4,746 (0.50) | 61 (1.04) | **hypertension** | | | |
| **Obesity** | | | | Yes | 21,186 (2.21) | 21,127 (2.22) | 59 (1.01) |
| Yes | 6,289 (0.66) | 6,245 (0.66) | 44 (0.75) | No | 931,627 (97.29) | 925,898 (97.28) | 5,729 (97.95) |
| No | 946,524 (98.84) | 940,780 (98.85) | 5,744 (98.20) | Unknown | 4,807 (0.50) | 4,746 (0.50) | 61 (1.04) |
| Unknown | 4,807 (0.50) | 4,746 (0.50) | 61 (1.04) |  |  |  |  |

| **Predictors** | **All births**  **N (%)** | **Livebirth**  **N (%)** | **Stillbirth**  **N (%)** | **Predictors** | **All births**  **N (%)** | **Livebirth**  **N (%)** | **Stillbirth**  **N (%)** |
| --- | --- | --- | --- | --- | --- | --- | --- |
| **Threatened miscarriage** | | | | **Urinary tract infection** | | | |
| Yes | 192,854 (20.14) | 188,994 (19.86) | 3,860 (65.99) | Yes | 50,630 (5.29) | 50,201 (5.27) | 429 (7.33) |
| No | 764,761 (79.86) | 762,775 (80.14) | 1,986 (33.95) | No | 906,985 (94.71) | 901,568 (94.73) | 5,417 (92.61) |
| Unknown | 5 (0.00) | 2 (0.00) | 3 (0.05) | Unknown | 5 (0.00) | 2 (0.00) | 3 (0.05) |
| **Preeclampsia** | | | | **Small-for-gestational**  **age** | | | |
| Yes | 61,161 (6.39) | 60,717 (6.38) | 444 (7.59) | Yes | 90,984 (9.50) | 88,860 (9.34) | 2,124 (36.31) |
| No | 896,454 (93.61) | 891,052 (93.62) | 5,402 (92.36) | No | 866,097 (90.44) | 862,461 (90.62) | 3,636 (62.16) |
| Unknown | 5 (0.00) | 2 (0.00) | 3 (0.05) | Unknown | 539 (0.06) | 450 (0.05) | 89 (1.52) |
| **Placenta praevia** | | | | **Cancer registration** | | | |
| Yes | 9,791 (1.02) | 9,641 (1.01) | 150 (2.56) | Yes | 2,877 (0.30) | 2,861 (0.30) | 16 (0.27) |
| No | 947,824 (98.98) | 942,128 (98.99) | 5,696 (97.38) | No | 949,936 (99.20) | 944,164 (99.20) | 5,772 (98.68) |
| Unknown | 5 (0.00) | 2 (0.00) | 3 (0.05) | Unknown | 4,807 (0.50) | 4,746 (0.50) | 61 (1.04) |
| **Pre-labour rupture of**  **membranes** | | | | **Threatened preterm**  **birth** | | | |
| Yes | 65,412 (6.83) | 64,389 (6.77) | 1,023 (17.49) | Yes | 14,178 (1.48) | 13,965 (1.47) | 213 (3.64) |
| No | 892,203 (93.17) | 887,380 (93.23) | 4,823 (82.46) | No | 943,369 (98.51) | 937,781 (98.53) | 5,588 (95.54) |
| Unknown | 5 (0.00) | 2 (0.00) | 3 (0.05) | Unknown | 73 (0.01) | 25 (0.00) | 48 (0.82) |
| **Unspecified antepartum**  **haemorrhage** | | | | **Plural** | | | |
| Yes | 33,589 (3.51) | 32,956 (3.46) | 633 (10.82) | Singleton | 930,953 (97.22) | 925,728 (97.26) | 5,225 (89.33) |
| No | 924,026 (96.49) | 918,813 (96.54) | 5,213 (89.13) | Twin | 25,716 (2.69) | 25,145 (2.64) | 571 (9.76) |
| Unknown | 5 (0.00) | 2 (0.00) | 3 (0.05) | Multiple gestation > 2 | 951 (0.10) | 898 (0.09) | 53 (0.91) |
| **Congenital anomaly** | | | |  |  |  |  |
| Yes | 51,087 (5.33) | 50,279 (5.28) | 808 (13.81) |  |  |  |  |
| No | 906,533 (94.67) | 901,492 (94.72) | 5,041 (86.19) |  |  |  |  |

**Supplementary Table 2c. Distribution of maternal characteristics for all births in Western Australia, 1980-2015: Previous pregnancy history**

| **PREVIOUS PREGNANCY HISTORY** | **All births N (%)** | **Livebirth N (%)** | **Stillbirth N (%)** | **Predictors** | **All births N (%)** | **Livebirth N (%)** | **Stillbirth N (%)** |
| --- | --- | --- | --- | --- | --- | --- | --- |
| **Previous stillbirth** | | | | **Previous threatened miscarriage** | | | |
| Nulliparous | 387,156 (40.43) | 384,605 (40.41) | 2,551 (43.61) | Nulliparous | 387,156 (40.43) | 384,605 (40.41) | 2,551 (43.61) |
| First birth, parity >0 | 102,681 (10.72) | 102,011 (10.72) | 670 (11.45) | First birth, parity >0 | 102,681 (10.72) | 102,011 (10.72) | 670 (11.45) |
| No history | 458,835 (47.91) | 456,387 (47.95) | 2,448 (41.85) | No history | 337,052 (35.20) | 335,360 (35.24) | 1,692 (28.93) |
| Yes, in earlier birth | 3,764 (0.39) | 3,713 (0.39) | 51 (0.87) | Yes, in earlier birth | 33,442 (3.49) | 33,212 (3.49) | 230 (3.93) |
| Yes, in last birth | 5,066 (0.53) | 4,952 (0.52) | 114 (1.95) | Yes, in last birth | 96,669 (10.09) | 95,966 (10.08) | 703 (12.02) |
| Unknown | 118 (0.01) | 103 (0.01) | 15 (0.26) | Unknown | 620 (0.06) | 617 (0.06) | 3 (0.05) |
| **Previous gestational diabetes** | | | | **Previous preeclampsia** | | | |
| Nulliparous | 387,156 (40.43) | 384,605 (40.41) | 2,551 (43.61) | Nulliparous | 387,156 (40.43) | 384,605 (40.41) | 2,551 (43.61) |
| First birth, parity >0 | 102,681 (10.72) | 102,011 (10.72) | 670 (11.45) | First birth, parity >0 | 102,681 (10.72) | 102,011 (10.72) | 670 (11.45) |
| No history | 452,658 (47.27) | 450,123 (47.29) | 2,535 (43.34) | No history | 417,857 (43.63) | 415,615 (43.67) | 2,242 (38.33) |
| Yes, in earlier birth | 2,178 (0.23) | 2,165 (0.23) | 13 (0.22) | Yes, in earlier birth | 14,747 (1.54) | 14,645 (1.54) | 102 (1.74) |
| Yes, in last birth | 12,305 (1.28) | 12,228 (1.28) | 77 (1.32) | Yes, in last birth | 34,538 (3.61) | 34,257 (3.60) | 281 (4.80) |
| Unknown | 642 (0.07) | 639 (0.07) | 3 (0.05) | Unknown | 641 (0.07) | 638 (0.07) | 3 (0.05) |
| **Previous gestational hypertension** | | | | **Previous placenta praevia** | | | |
| Nulliparous | 387,156 (40.43) | 384,605 (40.41) | 2,551 (43.61) | Nulliparous | 387,156 (40.43) | 384,605 (40.41) | 2,551 (43.61) |
| First birth, parity >0 | 102,681 (10.72) | 102,011 (10.72) | 670 (11.45) | First birth, parity >0 | 102,681 (10.72) | 102,011 (10.72) | 670 (11.45) |
| No history | 452,672 (47.27) | 450,145 (47.30) | 2,527 (43.20) | No history | 461,722 (48.22) | 459,144 (48.24) | 2,578 (44.08) |
| Yes, in earlier birth | 3,867 (0.40) | 3,842 (0.40) | 25 (0.43) | Yes, in earlier birth | 1,473 (0.15) | 1,461 (0.15) | 12 (0.21) |
| Yes, in last birth | 10,498 (1.10) | 10,437 (1.10) | 61 (1.04) | Yes, in last birth | 3,944 (0.41) | 3,909 (0.41) | 35 (0.60) |
| Unknown | 746 (0.08) | 731 (0.08) | 15 (0.26) | Unknown | 644 (0.07) | 641 (0.07) | 3 (0.05) |

Coding for previous conditions: nulliparous - parity 0; first birth, parity > 0 – no previous records exist because this was the first birth in the study period for a non-nulliparous mother; no history – previous birth records exist and there is no history of the condition; yes, in an earlier birth – the condition was present for an earlier birth; yes, in last birth – the condition was present for the most recent previous birth; unknown – unknown due to missing variables

| **Predictors** | **All births N (%)** | **Livebirth N (%)** | **Stillbirth N (%)** | **Predictors** | **All births N (%)** | **Livebirth N (%)** | **Stillbirth N (%)** |
| --- | --- | --- | --- | --- | --- | --- | --- |
| **Previous placental abruption** | | | | **Previous urinary tract infection** | | | |
| Nulliparous | 387,156 (40.43) | 384,605 (40.41) | 2,551 (43.61) | Nulliparous | 387,156 (40.43) | 384,605 (40.41) | 2,551 (43.61) |
| First birth, parity >0 | 102,681 (10.72) | 102,011 (10.72) | 670 (11.45) | First birth, parity >0 | 102,681 (10.72) | 102,011 (10.72) | 670 (11.45) |
| No history | 460,672 (48.11) | 458,147 (48.14) | 2,525 (43.17) | No history | 423,243 (44.20) | 420,970 (44.23) | 2,273 (38.86) |
| Yes, in earlier birth | 2,260 (0.24) | 2,233 (0.23) | 27 (0.46) | Yes, in earlier birth | 15,543 (1.62) | 15,389 (1.62) | 154 (2.63) |
| Yes, in last birth | 4,208 (0.44) | 4,135 (0.43) | 73 (1.25) | Yes, in last birth | 28,358 (2.96) | 28,160 (2.96) | 198 (3.39) |
| Unknown | 643 (0.07) | 640 (0.07) | 3 (0.05) | Unknown | 639 (0.07) | 636 (0.07) | 3 (0.05) |
| **Previous pre-labour rupture of membranes** | | | | **Previous threat preterm birth** | | | |
| Nulliparous | 387,156 (40.43) | 384,605 (40.41) | 2,551 (43.61) | Nulliparous | 387,156 (40.43) | 384,605 (40.41) | 2,551 (43.61) |
| First birth, parity >0 | 102,681 (10.72) | 102,011 (10.72) | 670 (11.45) | First birth, parity >0 | 102,681 (10.72) | 102,011 (10.72) | 670 (11.45) |
| No history | 420,302 (43.89) | 418,087 (43.93) | 2,215 (37.87) | No history | 459,171 (47.95) | 456,633 (47.98) | 2,538 (43.39) |
| Yes, in earlier birth | 13,824 (1.44) | 13,711 (1.44) | 113 (1.93) | Yes, in earlier birth | 2,363 (0.25) | 2,340 (0.25) | 23 (0.39) |
| Yes, in last birth | 33,025 (3.45) | 32,728 (3.44) | 297 (5.08) | Yes, in last birth | 5,638 (0.59) | 5,591 (0.59) | 47 (0.80) |
| Unknown | 632 (0.07) | 629 (0.07) | 3 (0.05) | Unknown | 611 (0.06) | 591 (0.06) | 20 (0.34) |
| **Previous unspecified antepartum haemorrhage** | | | | **Previous uterine rupture** | | | |
| Nulliparous | 387,156 (40.43) | 384,605 (40.41) | 2,551 (43.61) | Nulliparous | 387,156 (40.43) | 384,605 (40.41) | 2,551.0 (43.61) |
| First birth, parity >0 | 102,681 (10.72) | 102,011 (10.72) | 670 (11.45) | First birth, parity >0 | 102,681 (10.72) | 102,011 (10.72) | 670.0 (11.45) |
| No history | 442,759 (46.24) | 440,346 (46.27) | 2,413 (41.25) | No history | 466,999 (48.77) | 464,386 (48.79) | 2,613.0 (44.67) |
| Yes, in earlier birth | 7,994 (0.83) | 7,918 (0.83) | 76 (1.30) | Yes, in earlier birth | 11 (0.00) | 11 (0.00) | 0.0 (0.00) |
| Yes, in last birth | 16,396 (1.71) | 16,260 (1.71) | 136 (2.33) | Yes, in last birth | 27 (0.00) | 27 (0.00) | 0.0 (0.00) |
| Unknown | 634 (0.07) | 631 (0.07) | 3 (0.05) | Unknown | 746 (0.08) | 731 (0.08) | 15.0 (0.26) |

Coding for previous conditions: nulliparous - parity 0; first birth, parity > 0 – no previous records exist because this was the first birth in the study period for a non-nulliparous mother; no history – previous birth records exist and there is no history of the condition; yes, in an earlier birth – the condition was present for an earlier birth; yes, in last birth – the condition was present for the most recent previous birth; unknown – unknown due to missing variables

| **Predictors** | **All births N (%)** | **Livebirth N (%)** | **Stillbirth N (%)** | **Predictors** | **All births N (%)** | **Livebirth N (%)** | **Stillbirth N (%)** |
| --- | --- | --- | --- | --- | --- | --- | --- |
| **Previous cancer registry** | | | | **Previous caesarean delivery** | | | |
| Nulliparous | 387,156 (40.43) | 384,605 (40.41) | 2,551 (43.61) | Nulliparous | 387,156 (40.43) | 384,605 (40.41) | 2,551 (43.61) |
| First birth, parity >0 | 102,681 (10.72) | 102,011 (10.72) | 670 (11.45) | First birth, parity >0 | 102,681 (10.72) | 102,011 (10.72) | 670 (11.45) |
| No history | 465,604 (48.62) | 463,005 (48.65) | 2,599 (44.43) | No history | 360,342 (37.63) | 358,404 (37.66) | 1,938 (33.13) |
| Yes, in earlier birth | 370 (0.04) | 362 (0.04) | 8 (0.14) | Yes, in earlier birth | 6,783 (0.71) | 6,690 (0.70) | 93 (1.59) |
| Yes, in last birth | 1,063 (0.11) | 1,057 (0.11) | 6 (0.10) | Yes, in last birth | 100,152 (10.46) | 99,560 (10.46) | 592 (10.12) |
| Unknown | 746 (0.08) | 731 (0.08) | 15 (0.26) | Unknown | 506 (0.05) | 501 (0.05) | 5 (0.09) |
| **Previous small-for-gestational age** | | | | **Previous gestational age** | | | |
|  |  |  |  | Previous gestational age |  |  |  |
| Nulliparous | 387,156 (40.43) | 384,605 (40.41) | 2,551 (43.61) | <28 | 4,262 (0.45) | 4,131 (0.43) | 131 (2.24) |
| First birth, parity >0 | 102,681 (10.72) | 102,011 (10.72) | 670 (11.45) | 28≤previous gestational age≤31 | 3,343 (0.35) | 3,277 (0.34) | 66 (1.13) |
| No history | 394,093 (41.15) | 392,057 (41.19) | 2,036 (34.81) | 32≤previous gestational age≤36 | 28,691 (3.00) | 28,408 (2.98) | 283 (4.84) |
| Yes, in earlier birth | 21,580 (2.25) | 21,416 (2.25) | 164 (2.80) | 37≤previous gestational age | 429,341 (44.83) | 427,225 (44.89) | 2,116 (36.18) |
| Yes, in last birth | 49,784 (5.20) | 49,394 (5.19) | 390 (6.67) | Unknown | 491,983 (51.38) | 488,730 (51.35) | 3,253 (55.62) |
| Unknown | 2,326 (0.24) | 2,288 (0.24) | 38 (0.65) |  |  |  |  |
| **Previous birth defects** | | | |  |  |  |  |
| Yes | 38,832 (4.06) | 38,571 (4.05) | 261 (4.46) |  |  |  |  |
| No | 428,833 (44.78) | 426,481 (44.81) | 2,352 (40.21) |  |  |  |  |
| Unknown | 489,955 (51.16) | 486,719 (51.14) | 3,236 (55.33) |  |  |  |  |

Coding for previous conditions: nulliparous - parity 0; first birth, parity > 0 – no previous records exist because this was the first birth in the study period for a non-nulliparous mother; no history – previous birth records exist and there is no history of the condition; yes, in an earlier birth – the condition was present for an earlier birth; yes, in last birth – the condition was present for the most recent previous birth; unknown – unknown due to missing variables

# Supplementary Table 2d. Distribution of maternal characteristics for all births in Western Australia, 1980-2015: Parental birth outcomes

| **PARENTAL BIRTH OUTCOMES** |  |  |  |
| --- | --- | --- | --- |
| **Predictors** | **All births N (%)** | **Livebirth N (%)** | **Stillbirth N (%)** |
| **Parent small-for-gestational-age** | | | |
| Yes | 22,239 (2.32) | 22,097 (2.32) | 142 (2.43) |
| No | 115,029 (12.01) | 114,433 (12.02) | 596 (10.19) |
| Unknown | 820,352 (85.67) | 815,241 (85.66) | 5,111 (87.38) |
| **Parent preterm birth** | | | |
| Yes | 12,857 (1.34) | 12,772 (1.34) | 85 (1.45) |
| No | 124,570 (13.01) | 123,915 (13.02) | 655 (11.20) |
| Unknown | 820,193 (85.65) | 815,084 (85.64) | 5,109 (87.35) |
| **Parent congenital anomaly** | | | |
| Yes | 6,709 (0.70) | 6,672 (0.70) | 37 (0.63) |
| No | 132,910 (13.88) | 132,192 (13.89) | 718 (12.28) |
| Unknown | 818,001 (85.42) | 812,907 (85.41) | 5,094 (87.09) |

Parent – any parent listed on the birth record

# Supplementary Table 2e. Distribution of maternal characteristics for all births in Western Australia, 1980-2015: Maternal or paternal grandmothers pregnancy history

| **GRANDMOTHERS PREGNANCY HISTORY** | | | | | | | |
| --- | --- | --- | --- | --- | --- | --- | --- |
| **Predictors** | **All births N (%)** | **Livebirth N (%)** | **Stillbirth N (%)** | **Predictors** | **All births N (%)** | **Livebirth N (%)** | **Stillbirth N (%)** |
| **Grandmothers birth year** | | | | **Grandmothers circulatory system disease** | | | |
| 1980-1984 | 109,258 (11.41) | 108,409 (11.39) | 849 (14.52) | Yes | 1,268 (0.13) | 1,253 (0.13) | 15 (0.26) |
| 1985-1989 | 121,891 (12.73) | 121,038 (12.72) | 853 (14.58) | No | 137,737 (14.38) | 136,999 (14.39) | 738 (12.62) |
| 1995-1999 | 254,553 (26.58) | 252,974 (26.58) | 1,579 (27.00) | Unknown | 818,615 (85.48) | 813,519 (85.47) | 5,096 (87.13) |
| 2000-2004 | 124,910 (13.04) | 124,198 (13.05) | 712 (12.17) | **Grandmothers threatened miscarriage** | | | |
| 2005-2009 | 147,258 (15.38) | 146,466 (15.39) | 792 (13.54) | Yes | 35,227 (3.68) | 35,012 (3.68) | 215 (3.68) |
| 2010-2015 | 199,750 (20.86) | 198,686 (20.88) | 1,064 (18.19) | No | 104,372 (10.90) | 103,832 (10.91) | 540 (9.23) |
| **Grandmothers hypertension** | | | | Unknown | 818,021 (85.42) | 812,927 (85.41) | 5,094 (87.09) |
| Yes | 159 (0.02) | 156 (0.02) | 3 (0.05) | **Grandmothers preeclampsia** | | | |
| No | 139,438 (14.56) | 138,686 (14.57) | 752 (12.86) | Yes | 15,897 (1.66) | 15,808 (1.66) | 89 (1.52) |
| Unknown | 818,023 (85.42) | 812,929 (85.41) | 5,094 (87.09) | No | 123,702 (12.92) | 123,036 (12.93) | 666 (11.39) |
| **Grandmothers diabetes** | | | | Unknown | 818,021 (85.42) | 812,927 (85.41) | 5,094 (87.09) |
| Yes | 182 (0.02) | 181 (0.02) | 1 (0.02) | **Grandmothers placenta praevia** | | | |
| No | 139,415 (14.56) | 138,661 (14.57) | 754 (12.89) | Yes | 1,405 (0.15) | 1,398 (0.15) | 7 (0.12) |
| Unknown | 818,023 (85.42) | 812,929 (85.41) | 5,094 (87.09) | No | 138,192 (14.43) | 137,444 (14.44) | 748 (12.79) |
| **Grandmothers miscarriage** | | | | Unknown | 818,023 (85.42) | 812,929 (85.41) | 5,094 (87.09) |
| Yes | 4,482 (0.47) | 4,454 (0.47) | 28 (0.48) | **Grandmothers placental abruption** | | | |
| No | 134,523 (14.05) | 133,798 (14.06) | 725 (12.40) | Yes | 1,750 (0.18) | 1,742 (0.18) | 8 (0.14) |
| Unknown | 818,615 (85.48) | 813,519 (85.47) | 5,096 (87.13) | No | 137,847 (14.39) | 137,100 (14.40) | 747 (12.77) |
| **Grandmothers asthma** | | | | Unknown | 818,023 (85.42) | 812,929 (85.41) | 5,094 (87.09) |
| Yes | 2,090 (0.22) | 2,079 (0.22) | 11 (0.19) |  |  |  |  |
| No | 137,509 (14.36) | 136,765 (14.37) | 744 (12.72) |  |  |  |  |
| Unknown | 818,021 (85.42) | 812,927 (85.41) | 5,094 (87.09) |  |  |  |  |

| **Predictors** | **All births N (%)** | **Livebirth N (%)** | **Stillbirth N (%)** | **Predictors** | **All births N (%)** | **Livebirth N (%)** | **Stillbirth N (%)** |
| --- | --- | --- | --- | --- | --- | --- | --- |
| **Grandmothers pre-labour rupture of membranes** | | | | **Grandmothers cancer registry** | | | |
| Yes | 6,817 (0.71) | 6,772 (0.71) | 45 (0.77) | Yes | 151 (0.02) | 150 (0.02) | 1 (0.02) |
| No | 132,780 (13.87) | 132,070 (13.88) | 710 (12.14) | No | 138,853 (14.50) | 138,101 (14.51) | 752 (12.86) |
| Unknown | 818,023 (85.42) | 812,929 (85.41) | 5,094 (87.09) | Unknown | 818,616 (85.48) | 813,520 (85.47) | 5,096 (87.13) |
| **Grandmothers unspecified antepartum haemorrhage** | | | | **Grandmothers stillbirth** | | | |
| Yes | 5,315 (0.56) | 5,294 (0.56) | 21 (0.36) | Yes | 18 (0.00) | 18 (0.00) | 0.0 (0.00) |
| No | 134,282 (14.02) | 133,548 (14.03) | 734 (12.55) | No | 139,601 (14.58) | 138,846 (14.59) | 755.0 (12.91) |
| Unknown | 818,023 (85.42) | 812,929 (85.41) | 5,094 (87.09) | Unknown | 818,001 (85.42) | 812,907 (85.41) | 5,094.0 (87.09) |
| **Grandmothers gestational diabetes** | | | | **Grandmothers gestational hypertension** | | | |
| Yes | 1,179 (0.12) | 1,172 (0.12) | 7 (0.12) | Yes | 179 (0.02) | 178 (0.02) | 1 (0.02) |
| No | 138,418 (14.45) | 137,670 (14.46) | 748 (12.79) | No | 138,825 (14.50) | 138,073 (14.51) | 752 (12.86) |
| Unknown | 818,023 (85.42) | 812,929 (85.41) | 5,094 (87.09) | Unknown | 818,616 (85.48) | 813,520 (85.47) | 5,096 (87.13) |
| **Grandmothers urinary tract infection** | | | |  |  |  |  |
| Yes | 12,951 (1.35) | 12,847 (1.35) | 104 (1.78) |  |  |  |  |
| No | 126,646 (13.23) | 125,995 (13.24) | 651 (11.13) |  |  |  |  |
| Unknown | 818,023 (85.42) | 812,929 (85.41) | 5,094 (87.09) |  |  |  |  |

**Supplementary Table 3: Summary of classifier (GridSearchCV) parameters**

| **Classifiers** | ***GridSerchCV* parameters** |
| --- | --- |
| Logistic Regression | Inverse of L1 regularization strength: [0.001, 0.01, 0.03, 0.05, 0.07, 0.1] |
| Decision Tree | Quality of split measurement function: [gini, entropy] Maximum depth: [4, 6, 8, 10, 12, 14, 16]  Maximum predictors (fraction or constant): [sqrt, 0.50, 0.75, 0.85, all predictors] Minimum samples split (fractions): [0.0001, 0.001, 0.005, 0.01, 0.015, 0.02]  Minimum samples leaf (fractions): [0.0001, 0.001, 0.005, 0.01, 0.015, 0.02] |
| Random Forest | Quality of split measurement function: [gini, entropy] Maximum depth: [4, 6, 8, 10, 12, 14, 16]  Maximum predictors (fraction or constant): [sqrt, 0.50, 0.75, 0.85, all predictors] Minimum samples split (fractions): [0.0001, 0.001, 0.005, 0.01, 0.015, 0.02]  Minimum samples leaf (fractions): [0.0001, 0.001, 0.005, 0.01, 0.015, 0.02]  Number of trees in the forest: [50, 100, 300, 500, 700, 1000] |
| XGBoost | L1 regularization strength: [0, 0.001, 0.01, 0.05, 0.07, 0.1, 1]  L2 regularization strength: [0, 0.001, 0.01, 0.05, 0.07, 0.1, 1]  Learning rate: [0.001, 0.01, 0.03, 0.05, 0.1]  Maximum depth: [2, 3, 4, 5, 6, 7, 8]  Number of trees in the forest: [50, 100, 300, 500, 700, 1000]  Subsample: [0.3, 0.4, 0.5, 0.6, 0.7, 0.8] |
| Multi-layer Perceptron | Activation function: [relu, logistic, tanh] Solver: [sgd, adam]  L2 regularization strength: [0.01, 0.03, 0.05, 0.07, 0.1, 1]  Size of minibatches: [auto, 200, 500, 1500, 3000]  Number of neuron in the i^th^ hidden layer: [(all predictors, ), (all predictors, 0.5*all predictors), (100, ), (100, 50), (200,  ), (200, 100), (250, ), (250, 125)]  Learning rate: [0.0001, 0.001, 0.005, 0.01, 0.03, 0.05, 0.1] |

**Supplementary Table 4. Distribution of maternal characteristics by birth period: 1980-1989 (N=205,663)**

| **Predictors** | **1980-1989**  **N (%)** | **1990-1999**  **N (%)** | **2000-2009**  **N (%)** | **2010-2015**  **N (%)** |
| --- | --- | --- | --- | --- |
| **Total births** | **205,663(100)** | **228,883(100)** | **241,085(100)** | **199,750(100)** |
| **Livebirth** | **204,129(99.25)** | **227,439(99.37)** | **239,758(99.45)** | **198,686(99.47)** |
| **Stillbirth** | **1,534(0.75)** | **1,444(0.63)** | **1,327(0.55)** | **1,064(0.53)** |
| **SOCIO-DEMOGRAPHICS** | | | | |
| **Maternal Age** | | | | |
| <20 years | 13,883 (6.75) | 13,797 (6.03) | 12,957 (5.37) | 7,203 (3.61) |
| 20-24 years | 55,592 (27.03) | 44,305 (19.36) | 38,420 (15.94) | 28,303 (14.17) |
| 25-29 years | 79,391 (38.60) | 77,310 (33.78) | 66,893 (27.75) | 56,687 (28.38) |
| 30-34 years | 43,430 (21.12) | 65,366 (28.56) | 76,542 (31.75) | 65,450 (32.77) |
| 35-39 years | 11,731 (5.70) | 24,333 (10.63) | 38,929 (16.15) | 34,298 (17.17) |
| ≥40 years | 1,633 (0.79) | 3,772 (1.65) | 7,342 (3.05) | 7,806 (3.91) |
| Unknown | 3 (0.00) | 0 (0.00) | 2 (0.00) | 3 (0.00) |
| **Ethnicity** | | | | |
| Caucasian | 184,925 (89.92) | 198,834 (86.87) | 200,390 (83.12) | 144,756 (72.47) |
| Indigenous | 10,596 (5.15) | 13,333 (5.83) | 15,089 (6.26) | 5,081 (2.54) |
| Other | 10,141 (4.93) | 16,714 (7.30) | 25,603 (10.62) | 49,889 (24.98) |
| Unknown | 1 (0.00) | 2 (0.00) | 3 (0.00) | 24 (0.01) |
| **Socioeconomic status** | | | | |
| <20% (most disadvantaged) | 46,132 (22.43) | 49,405 (21.59) | 45,235 (18.76) | 28,831 (14.43) |
| 20-39% | 40,999 (19.94) | 47,617 (20.80) | 46,740 (19.39) | 31,958 (16.00) |
| 40-59% | 37,301 (18.14) | 46,011 (20.10) | 44,728 (18.55) | 38,907 (19.48) |
| 60-79% | 30,308 (14.74) | 37,929 (16.57) | 48,696 (20.20) | 48,029 (24.04) |
| >80% (least disadvantaged) | 32,164 (15.64) | 36,742 (16.05) | 48,761 (20.23) | 45,917 (22.99) |
| Unknown | 18,759 (9.12) | 11,179 (4.88) | 6,925 (2.87) | 6,108 (3.06) |
| **Urbanicity** | | | | |
| Urban | 126,577 (61.55) | 147,136 (64.28) | 160,292 (66.49) | 145,260 (72.72) |
| Rural | 46,635 (22.68) | 56,767 (24.80) | 61,334 (25.44) | 42,019 (21.04) |
| Unknown | 32,451 (15.78) | 24,980 (10.91) | 19,459 (8.07) | 12,471 (6.24) |
| **Birth year** | | | | |
| Birth year (5-6 year periods) | **1980-1984**: | **1990-1994**: | **2000-2004**: | **2010-2015**: |
|  | 109,258 (53.12) | 126,968 (55.47) | 124,910 (51.81) | 199,750 (100.00) |
|  | **1985-1989**: | **1995-1999**: | **2005-2009**: |  |

| 96,405 (46.88) | | 101,915 (44.53) | 116,175 (48.19) | |
| --- | --- | --- | --- | --- |
| **Smoking** | | | | |
| Yes | 1 (0.00) | 7,442 (3.25) | 43,817 (18.17) | 22,097 (11.06) |
| No | 69 (0.03) | 25,687 (11.22) | 197,255 (81.82) | 177,615 (88.92) |
| Unknown | 205,593 (99.97) | 195,754 (85.53) | 13 (0.01) | 38 (0.02) |
| **CHRONIC CONDITIONS** | | | | |
| **Essential hypertension** | | | | |
| Yes | 152 (0.07) | 222 (0.10) | 1,227 (0.51) | 1,239 (0.62) |
| No | 205,498 (99.92) | 228,660 (99.90) | 239,853 (99.49) | 198,504 (99.38) |
| Unknown | 13 (0.01) | 1 (0.00) | 5 (0.00) | 7 (0.00) |
| **Pre-existing diabetes** | | | | |
| Yes | 86 (0.04) | 752 (0.33) | 959 (0.40) | 1,069 (0.54) |
| No | 205,564 (99.95) | 228,130 (99.67) | 240,121 (99.60) | 198,674 (99.46) |
| Unknown | 13 (0.01) | 1 (0.00) | 5 (0.00) | 7 (0.00) |
| **Asthma** | | | | |
| Yes | 1,402 (0.68) | 6,864 (3.00) | 15,970 (6.62) | 13,859 (6.94) |
| No | 204,248 (99.31) | 222,018 (97.00) | 225,110 (93.37) | 185,884 (93.06) |
| Unknown | 13 (0.01) | 1 (0.00) | 5 (0.00) | 7 (0.00) |
| **Previous Miscarriage** | | | | |
| Yes | 3,611 (1.76) | 8,896 (3.89) | 7,092 (2.94) | 4,341 (2.17) |
| No | 200,873 (97.67) | 219,202 (95.77) | 233,077 (96.68) | 193,812 (97.03) |
| Unknown | 1,179 (0.57) | 785 (0.34) | 916 (0.38) | 1,597 (0.80) |
| **Obesity** | | | | |
| Yes | 0 (0.00) | 431 (0.19) | 2,752 (1.14) | 2,491 (1.25) |
| No | 204,484 (99.43) | 227,667 (99.47) | 237,417 (98.48) | 195,662 (97.95) |
| Unknown | 1,179 (0.57) | 785 (0.34) | 916 (0.38) | 1,597 (0.80) |
| **Circulatory system disease** | | | | |
| Yes | 1,080 (0.53) | 4,244 (1.85) | 2,100 (0.87) | 444 (0.22) |
| No | 203,404 (98.90) | 223,854 (97.80) | 238,069 (98.75) | 197,709 (98.98) |
| Unknown | 1,179 (0.57) | 785 (0.34) | 916 (0.38) | 1,597 (0.80) |
| **CURRENT PREGNANCY CHARACTERISTICS AND COMPLICATIONS** | | | | |
| **Parity** | | | | |
| 0 | 79,799 (38.80) | 89,907 (39.28) | 99,251 (41.17) | 84,983 (42.54) |
| 1 | 69,158 (33.63) | 76,457 (33.40) | 81,096 (33.64) | 68,524 (34.30) |
| 2 | 36,765 (17.88) | 38,904 (17.00) | 37,057 (15.37) | 29,332 (14.68) |

| >2  Unknown | 19,852 (9.65)  89 (0.04) | 23,613 (10.32)  2 (0.00) | 23,678 (9.82)  3 (0.00) | 16,887 (8.45)  24 (0.01) |
| --- | --- | --- | --- | --- |
| **Gestational diabetes** | | | | |
| Yes | 725 (0.35) | 5,439 (2.38) | 10,910 (4.53) | 22,831 (11.43) |
| No | 204,938 (99.65) | 223,444 (97.62) | 230,173 (95.47) | 176,916 (88.57) |
| Unknown | 0 (0.00) | 0 (0.00) | 0 (0.00) | 0 (0.00) |
| **Gestational hypertension** | | | | |
| Yes | 12 (0.01) | 2,498 (1.09) | 10,898 (4.52) | 5,874 (2.94) |
| No | 204,472 (99.42) | 225,600 (98.57) | 229,271 (95.10) | 192,279 (96.26) |
| Unknown | 1,179 (0.57) | 785 (0.34) | 916 (0.38) | 1,597 (0.80) |
| **Threatened miscarriage** | | | | |
| Yes | 39,671 (19.29) | 53,230 (23.26) | 54,251 (22.50) | 29,867 (14.95) |
| No | 165,992 (80.71) | 175,653 (76.74) | 186,832 (77.50) | 169,880 (85.05) |
| Unknown | 0 (0.00) | 0 (0.00) | 0 (0.00) | 0 (0.00) |
| **Preeclampsia** | | | | |
| Yes | 17,369 (8.45) | 18,681 (8.16) | 12,735 (5.28) | 7,181 (3.59) |
| No | 188,294 (91.55) | 210,202 (91.84) | 228,348 (94.72) | 192,566 (96.40) |
| Unknown | 0 (0.00) | 0 (0.00) | 0 (0.00) | 0 (0.00) |
| **Placenta praevia** | | | | |
| Yes | 1,704 (0.83) | 2,199 (0.96) | 2,895 (1.20) | 2,136 (1.07) |
| No | 203,959 (99.17) | 226,684 (99.04) | 238,188 (98.80) | 197,611 (98.93) |
| Unknown | 0 (0.00) | 0 (0.00) | 0 (0.00) | 0 (0.00) |
| **Pre-labour rupture of membranes** | | | | |
| Yes | 7,386 (3.59) | 13,086 (5.72) | 22,223 (9.22) | 16,369 (8.19) |
| No | 198,277 (96.41) | 215,797 (94.28) | 218,860 (90.78) | 183,378 (91.80) |
| Unknown | 0 (0.00) | 0 (0.00) | 0 (0.00) | 0 (0.00) |
| **Unspecified antepartum haemorrhage** | | | | |
| Yes | 5,573 (2.71) | 9,135 (3.99) | 8,927 (3.70) | 6,945 (3.48) |
| No | 200,090 (97.29) | 219,748 (96.01) | 232,156 (96.30) | 192,802 (96.52) |
| Unknown | 0 (0.00) | 0 (0.00) | 0 (0.00) | 0 (0.00) |
| **Urinary tract infection** | | | | |
| Yes | 10,215 (4.97) | 16,499 (7.21) | 12,195 (5.06) | 7,379 (3.69) |
| No | 195,448 (95.03) | 212,384 (92.79) | 228,888 (94.94) | 192,368 (96.30) |
| Unknown | 0 (0.00) | 0 (0.00) | 0 (0.00) | 0 (0.00) |
| **Small-for-gestational age** | | | | |

| Yes | 23,604 (11.48) | 22,354 (9.77) | 20,668 (8.57) | 16,762 (8.39) |
| --- | --- | --- | --- | --- |
| No | 181,811 (88.40) | 206,412 (90.18) | 220,339 (91.39) | 182,926 (91.58) |
| Unknown | 248 (0.12) | 117 (0.05) | 78 (0.03) | 62 (0.03) |
| **Cancer registration** | | | | |
| Yes | 174 (0.08) | 667 (0.29) | 1,032 (0.43) | 732 (0.37) |
| No | 204,310 (99.34) | 227,431 (99.37) | 239,137 (99.19) | 197,421 (98.83) |
| Unknown | 1,179 (0.57) | 785 (0.34) | 916 (0.38) | 1,597 (0.80) |
| **Threatened preterm birth** | | | | |
| Yes | 0 (0.00) | 868 (0.38) | 6,578 (2.73) | 5,193 (2.60) |
| No | 205,658 (100.00) | 228,000 (99.61) | 234,494 (97.27) | 194,519 (97.38) |
| Unknown | 5 (0.00) | 15 (0.01) | 13 (0.01) | 38 (0.02) |
| **Plural** | | | | |
| Singleton | 200,892 (97.68) | 222,492 (97.21) | 233,559 (96.88) | 194,190 (97.22) |
| Twin | 4,587 (2.23) | 6,106 (2.67) | 7,287 (3.02) | 5,446 (2.73) |
| Multiple gestation >2 | 184 (0.09) | 285 (0.12) | 239 (0.10) | 114 (0.06) |
| **Congenital anomaly** | | | | |
| Yes | 9,731 (4.73) | 13,289 (5.81) | 13,459 (5.58) | 10,043 (5.03) |
| No | 195,932 (95.27) | 215,594 (94.19) | 227,626 (94.42) | 189,707 (94.97) |
| **PREVIOUS PREGNANCY HISTORY** | | | | |
| **Previous stillbirth** | | | | |
| Nulliparous | 79,799 (38.80) | 89,907 (39.28) | 99,251 (41.17) | 84,983 (42.54) |
| First birth, parity >0 | 49,401 (24.02) | 15,509 (6.78) | 14,918 (6.19) | 16,621 (8.32) |
| No history | 74,848 (36.39) | 121,155 (52.93) | 124,547 (51.66) | 96,252 (48.19) |
| Yes, in earlier birth | 486 (0.24) | 1,015 (0.44) | 1,086 (0.45) | 820 (0.41) |
| Yes, in last birth | 1,040 (0.51) | 1,295 (0.57) | 1,280 (0.53) | 1,050 (0.53) |
| Unknown | 89 (0.04) | 2 (0.00) | 3 (0.00) | 24 (0.01) |
| **Previous gestational diabetes** | | | | |
| Nulliparous | 79,799 (38.80) | 89,907 (39.28) | 99,251 (41.17) | 84,983 (42.54) |
| First birth, parity >0 | 49,401 (24.02) | 15,509 (6.78) | 14,918 (6.19) | 16,621 (8.32) |
| No history | 76,204 (37.05) | 121,520 (53.09) | 121,715 (50.49) | 91,689 (45.90) |
| Yes, in earlier birth | 26 (0.01) | 233 (0.10) | 846 (0.35) | 901 (0.45) |
| Yes, in last birth | 131 (0.06) | 1,571 (0.69) | 4,196 (1.74) | 5,379 (2.69) |
| Unknown | 102 (0.05) | 143 (0.06) | 159 (0.07) | 177 (0.09) |
| **Previous gestational hypertension** | | | | |
| Nulliparous | 79,799 (38.80) | 89,907 (39.28) | 99,251 (41.17) | 84,983 (42.54) |

| First birth, parity >0 | 49,401 (24.02) | 15,509 (6.78) | 14,918 (6.19) | 16,621 (8.32) |
| --- | --- | --- | --- | --- |
| No history | 76,275 (37.09) | 122,495 (53.52) | 119,693 (49.65) | 92,722 (46.42) |
| Yes, in earlier birth | 1 (0.00) | 120 (0.05) | 1,613 (0.67) | 1,767 (0.88) |
| Yes, in last birth | 7 (0.00) | 707 (0.31) | 5,450 (2.26) | 3,457 (1.73) |
| Unknown | 180 (0.09) | 145 (0.06) | 160 (0.07) | 200 (0.10) |
| **Previous threatened miscarriage** | | | | |
| Nulliparous | 79,799 (38.80) | 89,907 (39.28) | 99,251 (41.17) | 84,983 (42.54) |
| First birth, parity >0 | 49,401 (24.02) | 15,509 (6.78) | 14,918 (6.19) | 16,621 (8.32) |
| No history | 59,403 (28.88) | 87,051 (38.03) | 88,132 (36.56) | 71,839 (35.96) |
| Yes, in earlier birth | 2,535 (1.23) | 9,007 (3.94) | 10,640 (4.41) | 7,825 (3.92) |
| Yes, in last birth | 14,425 (7.01) | 27,270 (11.91) | 27,993 (11.61) | 18,308 (9.17) |
| Unknown | 100 (0.05) | 139 (0.06) | 151 (0.06) | 174 (0.09) |
| **Previous preeclampsia** | | | | |
| Nulliparous | 79,799 (38.80) | 89,907 (39.28) | 99,251 (41.17) | 84,983 (42.54) |
| First birth, parity >0 | 49,401 (24.02) | 15,509 (6.78) | 14,918 (6.19) | 16,621 (8.32) |
| No history | 67,696 (32.92) | 107,857 (47.12) | 113,125 (46.92) | 91,163 (45.64) |
| Yes, in earlier birth | 1,480 (0.72) | 4,688 (2.05) | 4,641 (1.93) | 2,464 (1.23) |
| Yes, in last birth | 7,188 (3.50) | 10,779 (4.71) | 8,991 (3.73) | 4,340 (2.17) |
| Unknown | 99 (0.05) | 143 (0.06) | 159 (0.07) | 179 (0.09) |
| **Previous placenta praevia** | | | | |
| Nulliparous | 79,799 (38.80) | 89,907 (39.28) | 99,251 (41.17) | 84,983 (42.54) |
| First birth, parity >0 | 49,401 (24.02) | 15,509 (6.78) | 14,918 (6.19) | 16,621 (8.32) |
| No history | 75,717 (36.82) | 122,001 (53.30) | 125,037 (51.86) | 96,722 (48.42) |
| Yes, in earlier birth | 123 (0.06) | 399 (0.17) | 481 (0.20) | 346 (0.17) |
| Yes, in last birth | 521 (0.25) | 924 (0.40) | 1,239 (0.51) | 899 (0.45) |
| Unknown | 102 (0.05) | 143 (0.06) | 159 (0.07) | 179 (0.09) |
| **Previous placental abruption** | | | | |
| Nulliparous | 79,799 (38.80) | 89,907 (39.28) | 99,251 (41.17) | 84,983 (42.54) |
| First birth, parity >0 | 49,401 (24.02) | 15,509 (6.78) | 14,918 (6.19) | 16,621 (8.32) |
| No history | 75,188 (36.56) | 121,319 (53.00) | 125,139 (51.91) | 96,927 (48.52) |
| Yes, in earlier birth | 271 (0.13) | 788 (0.34) | 605 (0.25) | 356 (0.18) |
| Yes, in last birth | 902 (0.44) | 1,218 (0.53) | 1,013 (0.42) | 684 (0.34) |
| Unknown | 102 (0.05) | 142 (0.06) | 159 (0.07) | 179 (0.09) |
| **Previous pre-labour rupture of membranes** | | | | |
| Nulliparous | 79,799 (38.80) | 89,907 (39.28) | 99,251 (41.17) | 84,983 (42.54) |

| First birth, parity >0 | 49,401 (24.02) | 15,509 (6.78) | 14,918 (6.19) | 16,621 (8.32) |
| --- | --- | --- | --- | --- |
| No history | 73,052 (35.52) | 114,350 (49.96) | 110,869 (45.99) | 83,714 (41.91) |
| Yes, in earlier birth | 640 (0.31) | 2,686 (1.17) | 4,657 (1.93) | 4,551 (2.28) |
| Yes, in last birth | 2,669 (1.30) | 6,289 (2.75) | 11,233 (4.66) | 9,709 (4.86) |
| Unknown | 102 (0.05) | 142 (0.06) | 157 (0.07) | 172 (0.09) |
| **Previous unspecified antepartum haemorrhage** | | | | |
| Nulliparous | 79,799 (38.80) | 89,907 (39.28) | 99,251 (41.17) | 84,983 (42.54) |
| First birth, parity >0 | 49,401 (24.02) | 15,509 (6.78) | 14,918 (6.19) | 16,621 (8.32) |
| No history | 74,142 (36.05) | 116,450 (50.88) | 119,203 (49.44) | 92,607 (46.36) |
| Yes, in earlier birth | 454 (0.22) | 2,099 (0.92) | 2,765 (1.15) | 1,871 (0.94) |
| Yes, in last birth | 1,766 (0.86) | 4,777 (2.09) | 4,791 (1.99) | 3,493 (1.75) |
| Unknown | 101 (0.05) | 141 (0.06) | 157 (0.07) | 175 (0.09) |
| **Previous urinary tract infection** | | | | |
| Nulliparous | 79,799 (38.80) | 89,907 (39.28) | 99,251 (41.17) | 84,983 (42.54) |
| First birth, parity >0 | 49,401 (24.02) | 15,509 (6.78) | 14,918 (6.19) | 16,621 (8.32) |
| No history | 71,601 (34.81) | 110,512 (48.28) | 112,653 (46.73) | 90,143 (45.13) |
| Yes, in earlier birth | 957 (0.47) | 4,155 (1.82) | 5,676 (2.35) | 3,219 (1.61) |
| Yes, in last birth | 3,803 (1.85) | 8,658 (3.78) | 8,431 (3.50) | 4,606 (2.31) |
| Unknown | 102 (0.05) | 142 (0.06) | 156 (0.06) | 178 (0.09) |
| **Previous threat preterm birth** | | | | |
| Nulliparous | 79,799 (38.80) | 89,907 (39.28) | 99,251 (41.17) | 84,983 (42.54) |
| First birth, parity >0 | 49,401 (24.02) | 15,509 (6.78) | 14,918 (6.19) | 16,621 (8.32) |
| No history | 76,234 (37.07) | 123,335 (53.89) | 123,074 (51.05) | 94,505 (47.31) |
| Yes, in earlier birth | 0 (0.00) | 0 (0.00) | 918 (0.38) | 1,239 (0.62) |
| Yes, in last birth | 0 (0.00) | 4 (0.00) | 2,812 (1.17) | 2,319 (1.16) |
| Unknown | 229 (0.11) | 128 (0.06) | 112 (0.05) | 83 (0.04) |
| **Previous uterine rupture** | | | | |
| Nulliparous | 79,799 (38.80) | 89,907 (39.28) | 99,251 (41.17) | 84,983 (42.54) |
| First birth, parity >0 | 49,401 (24.02) | 15,509 (6.78) | 14,918 (6.19) | 16,621 (8.32) |
| No history | 76,276 (37.09) | 123,315 (53.88) | 126,744 (52.57) | 97,937 (49.03) |
| Yes, in earlier birth | 2 (0.00) | 0 (0.00) | 3 (0.00) | 4 (0.00) |
| Yes, in last birth | 5 (0.00) | 7 (0.00) | 9 (0.00) | 5 (0.00) |
| Unknown | 180 (0.09) | 145 (0.06) | 160 (0.07) | 200 (0.10) |
| **Previous cancer registration** | | | | |
| Nulliparous | 79,799 (38.80) | 89,907 (39.28) | 99,251 (41.17) | 84,983 (42.54) |

| First birth, parity >0 | 49,401 (24.02) | 15,509 (6.78) | 14,918 (6.19) | 16,621 (8.32) |
| --- | --- | --- | --- | --- |
| No history | 76,236 (37.07) | 123,064 (53.77) | 126,195 (52.34) | 97,521 (48.82) |
| Yes, in earlier birth | 5 (0.00) | 67 (0.03) | 145 (0.06) | 115 (0.06) |
| Yes, in last birth | 42 (0.02) | 191 (0.08) | 416 (0.17) | 310 (0.16) |
| Unknown | 180 (0.09) | 145 (0.06) | 160 (0.07) | 200 (0.10) |
| **Previous small-for-gestational age** | | | | |
| Nulliparous | 79,799 (38.80) | 89,907 (39.28) | 99,251 (41.17) | 84,983 (42.54) |
| First birth, parity >0 | 49,401 (24.02) | 15,509 (6.78) | 14,918 (6.19) | 16,621 (8.32) |
| No history | 62,998 (30.63) | 102,581 (44.82) | 107,648 (44.65) | 84,832 (42.47) |
| Yes, in earlier birth | 2,168 (1.05) | 6,542 (2.86) | 6,575 (2.73) | 4,175 (2.09) |
| Yes, in last birth | 9,771 (4.75) | 14,013 (6.12) | 12,504 (5.19) | 9,001 (4.51) |
| Unknown | 1,526 (0.74) | 331 (0.14) | 189 (0.08) | 138 (0.07) |
| **Previous congenital anomalies** | | | | |
| Yes | 4,851 (2.36) | 10,496 (4.59) | 11,561 (4.80) | 8,221 (4.12) |
| No | 71,523 (34.78) | 112,969 (49.36) | 115,352 (47.85) | 89,901 (45.01) |
| Unknown | 129,289 (62.86) | 105,418 (46.06) | 114,172 (47.36) | 101,628 (50.88) |
| **Previous caesarean delivery** | | | | |
| Nulliparous | 79,799 (38.80) | 89,907 (39.28) | 99,251 (41.17) | 84,983 (42.54) |
| First birth, parity >0 | 49,401 (24.02) | 15,509 (6.78) | 14,918 (6.19) | 16,621 (8.32) |
| No history | 66,086 (32.13) | 99,972 (43.68) | 93,511 (38.79) | 67,761 (33.92) |
| Yes, in earlier birth | 508 (0.25) | 2,016 (0.88) | 2,064 (0.86) | 1,500 (0.75) |
| Yes, in last birth | 9,727 (4.73) | 21,349 (9.33) | 31,229 (12.95) | 28,826 (14.43) |
| Unknown | 142 (0.07) | 130 (0.06) | 112 (0.05) | 59 (0.03) |
| **Previous gestational age** | | | | |
| previous gestational age<28 | 753 (0.37) | 1,002 (0.44) | 1,137 (0.47) | 1,015 (0.51) |
| 28≤previous gestational age≤31 | 623 (0.30) | 869 (0.38) | 868 (0.36) | 662 (0.33) |
| 32≤previous gestational age≤36 | 4,309 (2.10) | 7,498 (3.28) | 8,094 (3.36) | 6,127 (3.07) |
| previous gestational age≥37 | 69,298 (33.69) | 113,840 (49.74) | 116,658 (48.39) | 90,228 (45.17) |
| Unknown | 130,680 (63.54) | 105,674 (46.17) | 114,328 (47.42) | 101,718 (50.92) |
| **PARENTAL BIRTH OUTCOMES** | | | | |
| **Parents small-for-gestational-age** | | | | |
| Yes | 0 (0.00) | 195 (0.09) | 7,001 (2.90) | 13,193 (6.60) |
| No | 0 (0.00) | 789 (0.34) | 33,545 (13.91) | 71,660 (35.87) |
| Unknown | 205,663 (100.00) | 227,899 (99.57) | 200,539 (83.18) | 114,897 (57.52) |
| **Parents preterm birth** | | | | |

| Yes | 0 (0.00) | 74 (0.03) | 3,740 (1.55) | 8,025 (4.02) |
| --- | --- | --- | --- | --- |
| No | 0 (0.00) | 912 (0.40) | 36,869 (15.29) | 76,904 (38.50) |
| Unknown | 205,663 (100.00) | 227,897 (99.57) | 200,476 (83.16) | 114,821 (57.48) |
| **Parents congenital anomaly** | | | | |
| Yes | 0 (0.00) | 25 (0.01) | 1,762 (0.73) | 4,428 (2.22) |
| No | 0 (0.00) | 1,048 (0.46) | 39,803 (16.51) | 81,422 (40.76) |
| Unknown | 205,663 (100.00) | 227,810 (99.53) | 199,520 (82.76) | 113,900 (57.02) |
| **GRANDMOTHERS PREGNANCY HISTORY** | | | | |
| **Grandmothers birth year** | | | | |
| Birth year (5-6 year periods) | **1980-1984**: | **1995-1999**: | **2000-2004**: | **2010-2015**: |
|  | 109,258 (53.12) | 228,883 (100.00) | 124,910 (51.81) | 199,750 (100.00) |
|  | **1985-1989** : |  | **2005-2009**: |  |
|  | 96,405 (46.88) |  | 116,175 (48.19) |  |
| **Grandmothers hypertension** | | | | |
| Yes | 0 (0.00) | 0 (0.00) | 45 (0.02) | 103 (0.05) |
| No | 0 (0.00) | 1,073 (0.47) | 41,513 (17.22) | 85,734 (42.92) |
| Unknown | 205,663 (100.00) | 227,810 (99.53) | 199,527 (82.76) | 113,913 (57.03) |
| **Grandmothers diabetes** | | | | |
| Yes | 0 (0.00) | 0 (0.00) | 43 (0.02) | 130 (0.07) |
| No | 0 (0.00) | 1,073 (0.47) | 41,515 (17.22) | 85,707 (42.91) |
| Unknown | 205,663 (100.00) | 227,810 (99.53) | 199,527 (82.76) | 113,913 (57.03) |
| **Grandmothers miscarriage** | | | | |
| Yes | 0 (0.00) | 2 (0.00) | 961 (0.40) | 3,220 (1.61) |
| No | 0 (0.00) | 1,064 (0.46) | 40,402 (16.76) | 82,270 (41.19) |
| Unknown | 205,663 (100.00) | 227,817 (99.53) | 199,722 (82.84) | 114,260 (57.20) |
| **Grandmothers asthma** | | | | |
| Yes | 0 (0.00) | 4 (0.00) | 402 (0.17) | 1,555 (0.78) |
| No | 0 (0.00) | 1,069 (0.47) | 41,157 (17.07) | 84,283 (42.19) |
| Unknown | 205,663 (100.00) | 227,810 (99.53) | 199,526 (82.76) | 113,912 (57.03) |
| **Grandmothers circulatory system disease** |  |  |  |  |
| Yes | 0 (0.00) | 2 (0.00) | 312 (0.13) | 879 (0.44) |
| No | 0 (0.00) | 1,064 (0.46) | 41,050 (17.03) | 84,612 (42.36) |
| Unknown | 205,663 (100.00) | 227,817 (99.53) | 199,723 (82.84) | 114,259 (57.20) |
| **Grandmothers threatened miscarriage** | | | | |
| Yes | 0 (0.00) | 203 (0.09) | 9,913 (4.11) | 22,327 (11.18) |
| No | 0 (0.00) | 870 (0.38) | 31,646 (13.13) | 63,511 (31.80) |

| Unknown | 205,663 (100.00) | 227,810 (99.53) | 199,526 (82.76) | 113,912 (57.03) |
| --- | --- | --- | --- | --- |
| **Grandmothers preeclampsia** | | | | |
| Yes | 0 (0.00) | 96 (0.04) | 4,566 (1.89) | 9,966 (4.99) |
| No | 0 (0.00) | 977 (0.43) | 36,994 (15.34) | 75,871 (37.98) |
| Unknown | 205,663 (100.00) | 227,810 (99.53) | 199,525 (82.76) | 113,913 (57.03) |
| **Grandmothers placenta praevia** | | | | |
| Yes | 0 (0.00) | 4 (0.00) | 419 (0.17) | 876 (0.44) |
| No | 0 (0.00) | 1,069 (0.47) | 41,139 (17.06) | 84,961 (42.53) |
| Unknown | 205,663 (100.00) | 227,810 (99.53) | 199,527 (82.76) | 113,913 (57.03) |
| **Grandmothers placental abruption** | | | | |
| Yes | 0 (0.00) | 7 (0.00) | 541 (0.22) | 1,050 (0.53) |
| No | 0 (0.00) | 1,066 (0.47) | 41,017 (17.01) | 84,787 (42.45) |
| Unknown | 205,663 (100.00) | 227,810 (99.53) | 199,527 (82.76) | 113,913 (57.03) |
| **Grandmothers pre-labour rupture of membranes** | | | | |
| Yes | 0 (0.00) | 51 (0.02) | 1,896 (0.79) | 4,329 (2.17) |
| No | 0 (0.00) | 1,022 (0.45) | 39,662 (16.45) | 81,508 (40.81) |
| Unknown | 205,663 (100.00) | 227,810 (99.53) | 199,527 (82.76) | 113,913 (57.03) |
| **Grandmothers unspecified antepartum haemorrhage** | | | | |
| Yes | 0 (0.00) | 17 (0.01) | 1,434 (0.59) | 3,435 (1.72) |
| No | 0 (0.00) | 1,056 (0.46) | 40,124 (16.64) | 82,402 (41.25) |
| Unknown | 205,663 (100.00) | 227,810 (99.53) | 199,527 (82.76) | 113,913 (57.03) |
| **Grandmothers gestational diabetes** | | | | |
| Yes | 0 (0.00) | 5 (0.00) | 239 (0.10) | 849 (0.43) |
| No | 0 (0.00) | 1,068 (0.47) | 41,319 (17.14) | 84,988 (42.55) |
| Unknown | 205,663 (100.00) | 227,810 (99.53) | 199,527 (82.76) | 113,913 (57.03) |
| **Grandmothers urinary tract infection** | | | | |
| Yes | 0 (0.00) | 125 (0.05) | 4,144 (1.72) | 7,654 (3.83) |
| No | 0 (0.00) | 948 (0.41) | 37,414 (15.52) | 78,183 (39.14) |
| Unknown | 205,663 (100.00) | 227,810 (99.53) | 199,527 (82.76) | 113,913 (57.03) |
| **Grandmothers cancer registry** | | | | |
| Yes | 0 (0.00) | 0 (0.00) | 28 (0.01) | 113 (0.06) |
| No | 0 (0.00) | 1,066 (0.47) | 41,334 (17.14) | 85,377 (42.74) |
| Unknown | 205,663 (100.00) | 227,817 (99.53) | 199,723 (82.84) | 114,260 (57.20) |
| **Grandmothers history of stillbirth** | | | | |
| Yes | 0 (0.00) | 2 (0.00) | 9 (0.00) | 6 (0.00) |

| No Unknown | 0 (0.00)  205,663 (100.00) | 1,071 (0.47)  227,810 (99.53) | 41,556 (17.24)  199,520 (82.76) | 85,844 (42.98)  113,900 (57.02) |
| --- | --- | --- | --- | --- |
| **Grandmothers gestational hypertension** | | | | |
| Yes | 0 (0.00) | 0 (0.00) | 8 (0.00) | 167 (0.08) |
| No | 0 (0.00) | 1,066 (0.47) | 41,354 (17.15) | 85,323 (42.71) |
| Unknown | 205,663 (100.00) | 227,817 (99.53) | 199,723 (82.84) | 114,260 (57.20) |

Coding for previous conditions: nulliparous - parity 0; first birth, parity > 0 – no previous records exist because this was the first birth in the study period for a non-nulliparous mother; no history – previous birth records exist and there is no history of the condition; yes, in an earlier birth – the condition was present for an earlier birth; yes, in last birth – the condition was present for the most recent previous birth; unknown – unknown due to missing variables

Grandparent – maternal or paternal grandparent Parent – any parent listed on the birth record

# Supplementary Table 5. Sensitivity analyses: Excluded multiples (A1), excluded congenital anomalies (A2), excluded births before 2000 (A3), excluded early preterm births <28 weeks of gestation (A4). All models use socio-demographics, chronic conditions, and current pregnancy characteristics and complications as predictors (Model A).

| **Classifiers** | **Model** | **AUC (95% CI)** | **+LR (95% CI)** | **-LR (95% CI)** | **5% FPR** | | | |
| --- | --- | --- | --- | --- | --- | --- | --- | --- |
|  |  |  |  |  | **Sensitivity (%, 95% CI)** | **PPV (%, 95% CI)** | **NPV (%, 95% CI)** | **Correctly Classified (%, 95% CI)** |
| Logistic Regression | A1 | 0.825 (0.819-0.831) | 8.015 (7.649-8.382) | 0.631 (0.612-0.650) | 40.074 (38.243-41.905) | 4.302 (4.114-4.490) | 99.647 (99.637-99.658) | 94.694 (94.683-94.704) |
| Logistic Regression | A2 | 0.826 (0.820-0.831) | 8.023 (7.674-8.371) | 0.630 (0.612-0.649) | 40.118 (38.372-41.864) | 4.673 (4.480-4.866) | 99.616 (99.605-99.627) | 94.666 (94.656-94.677) |
| Logistic Regression | A3 | 0.813 (0.802-0.824) | 7.539 (7.134-7.943) | 0.656 (0.635-0.677) | 37.663 (35.655-39.671) | 3.877 (3.678-4.076) | 99.650 (99.639-99.661) | 94.698 (94.685-94.712) |
| Logistic Regression | A4 | 0.801 (0.793-0.809) | 7.248 (6.894-7.601) | 0.671 (0.653-0.690) | 36.245 (34.473-38.017) | 2.446 (2.331-2.562) | 99.768 (99.762-99.775) | 94.797 (94.790-94.803) |
| Decision Tree | A1 | 0.816 (0.810-0.822) | 8.006 (7.696-8.316) | 0.642 (0.627-0.657) | 38.874 (37.422-40.326) | 4.298 (4.139-4.457) | 99.641 (99.633-99.649) | 94.825 (94.680-94.970) |
| Decision Tree | A2 | 0.816 (0.811-0.822) | 7.984 (7.521-8.447) | 0.630 (0.615-0.645) | 40.170 (38.771-41.568) | 4.651 (4.395-4.907) | 99.616 (99.607-99.626) | 94.616 (94.434-94.798) |
| Decision Tree | A3 | 0.792 (0.779-0.806) | 7.592 (7.166-8.019) | 0.658 (0.639-0.677) | 37.433 (35.613-39.252) | 3.903 (3.692-4.115) | 99.649 (99.639-99.659) | 94.757 (94.653-94.860) |
| Decision Tree | A4 | 0.797 (0.789-0.805) | 7.891 (7.336-8.446) | 0.641 (0.618-0.665) | 39.063 (36.864-41.261) | 2.657 (2.475-2.839) | 99.779 (99.771-99.787) | 94.844 (94.726-94.961) |
| Random Forest | A1 | 0.825 (0.819-0.831) | 8.037 (7.708-8.365) | 0.630 (0.612-0.647) | 40.190 (38.548-41.832) | 4.313 (4.145-4.482) | 99.648 (99.638-99.658) | 94.693 (94.683-94.703) |
| Random Forest | A2 | 0.826 (0.821-0.832) | 7.953 (7.622-8.284) | 0.634 (0.617-0.651) | 39.755 (38.104-41.406) | 4.634 (4.451-4.817) | 99.614 (99.603-99.625) | 94.665 (94.655-94.676) |
| Random Forest | A3 | 0.810 (0.800-0.820) | 7.432 (7.079-7.785) | 0.661 (0.643-0.680) | 37.168 (35.402-38.934) | 3.824 (3.650-3.999) | 99.647 (99.638-99.657) | 94.691 (94.680-94.702) |
| Random Forest | A4 | 0.806 (0.800-0.813) | 7.732 (7.307-8.157) | 0.645 (0.623-0.668) | 38.665 (36.544-40.786) | 2.605 (2.466-2.745) | 99.777 (99.769-99.785) | 94.805 (94.797-94.813) |
| XGBoost | A1 | 0.834 (0.827-0.840) | 8.575 (8.278-8.871) | 0.602 (0.586-0.617) | 42.860 (41.379-44.342) | 4.589 (4.438-4.739) | 99.664 (99.655-99.672) | 94.710 (94.702-94.719) |
| XGBoost | A2 | 0.836 (0.830-0.841) | 8.747 (8.373-9.121) | 0.592 (0.573-0.612) | 43.729 (41.853-45.605) | 5.074 (4.869-5.279) | 99.639 (99.627-99.651) | 94.689 (94.679-94.700) |
| XGBoost | A3 | 0.817 (0.805-0.828) | 8.136 (7.694-8.578) | 0.625 (0.602-0.648) | 40.623 (38.436-42.810) | 4.171 (3.954-4.388) | 99.667 (99.654-99.679) | 94.717 (94.702-94.732) |
| XGBoost | A4 | 0.810 (0.803-0.818) | 8.002 (7.511-8.494) | 0.632 (0.606-0.657) | 40.011 (37.553-42.470) | 2.694 (2.532-2.856) | 99.782 (99.773-99.791) | 94.810 (94.802-94.819) |
| MLPs | A1 | 0.829 (0.823-0.835) | 8.370 (8.070-8.669) | 0.612 (0.596-0.628) | 41.854 (40.359-43.349) | 4.484 (4.331-4.637) | 99.658 (99.649-99.667) | 94.703 (94.694-94.711) |
| MLPs | A2 | 0.832 (0.827-0.837) | 8.429 (8.099-8.759) | 0.609 (0.591-0.626) | 42.157 (40.510-43.803) | 4.898 (4.717-5.080) | 99.629 (99.619-99.640) | 94.678 (94.667-94.688) |
| MLPs | A3 | 0.816 (0.805-0.827) | 8.018 (7.642-8.395) | 0.630 (0.610-0.650) | 40.092 (38.201-41.983) | 4.114 (3.929-4.298) | 99.664 (99.653-99.674) | 94.708 (94.699-94.717) |
| MLPs | A4 | 0.799 (0.791-0.806) | 7.693 (7.228-8.157) | 0.648 (0.623-0.672) | 38.481 (36.163-40.799) | 2.592 (2.440-2.745) | 99.776 (99.768-99.785) | 94.803 (94.794-94.811) |

All models use socio-demographics, chronic conditions, current pregnancy complications and other characteristics as predictors (Model A). A1 – Restricted to singletons.

A2 – Restricted to births without congenital anomalies.

A3 – Restricted to births from the year 2000, when most predictors were available. A4 – Restricted to births from 28 weeks gestation.

Abbreviations: AUC – Area under the receiving-operator characteristic curve; +LR – Positive likelihood ratio; -LR – Negative likelihood ratio; FPR – alpha (type I error) = 1-specificity; Sensitivity – detection rate, TPR; TP – True Positives; FP – False Positives; TN – True Negatives; FN – False Negatives; PPV – Positive predictive value = TP/(TP+FP); NPV - Negative predictive value = TN/(FN+TN); CI – Confidence Interval.

**Supplementary Table 6: Performance of models, including 95% confidence intervals, for predicting stillbirth using different classification algorithms and 10-fold cross validation.**

| **Classifiers** | **Mod el** | **AUC (95% CI)** | **5% FPR** | | | | | | **10% FPR** | | | | | |
| --- | --- | --- | --- | --- | --- | --- | --- | --- | --- | --- | --- | --- | --- | --- |
|  |  |  | **+LR (95% CI)** | **-LR (95% CI)** | **Sensitivity (%, 95% CI)** | **PPV (%, 95%**  **CI)** | **NPV (%, 95% CI)** | **Correctly Classified (%, 95% CI)** | **+LR (95% CI)** | **-LR (95% CI)** | **Sensitivity (%, 95% CI)** | **PPV (%, 95%**  **CI)** | **NPV (%, 95% CI)** | **Correctly Classified**  **(%, 95% CI)** |
|  | A | 0.830  (0.825-  0.836) | 8.10  (7.77-  8.43) | 0.63  (0.61-  0.64) | 40.5 (38.9-  42.2) | 4.72 (4.54-  4.90) | 99.62  (99.61-  99.63) | 94.67 (94.66-  94.68) | 5.52  (5.40-  5.64) | 0.50  (0.49-  0.51) | 55.2 (54.0-  56.4) | 3.26 (3.19-  3.33) | 99.7  (99.69-  99.70) | 89.79 (89.78-  89.80) |
|  | B | 0.834  (0.826-  0.843) | 8.07  (7.57-  8.58) | 0.63  (0.60-  0.65) | 40.5 (38.0-  42.9) | 4.32 (4.07-  4.58) | 99.65  (99.64-  99.67) | 94.68 (94.64-  94.72) | 5.57  (5.39-  5.75) | 0.49  (0.47-  0.51) | 55.7 (54.0-  57.4) | 3.02 (2.93-  3.12) | 99.73  (99.72-  99.74) | 89.80 (89.77-  89.84) |
| Logistic | C | 0.811  (0.797-  0.824) | 7.59  (7.04-  8.13) | 0.66  (0.63-  0.68) | 37.8 (35.0-  40.5) | 3.89 (3.62-  4.16) | 99.65  (99.64-  99.67) | 94.72 (94.65-  94.79) | 5.14  (4.92-  5.36) | 0.54  (0.51-  0.56) | 51.6 (49.4-  53.8) | 2.67 (2.56-  2.77) | 99.71  (99.70-  99.73) | 89.75 (89.68-  89.83) |
| Regression | D | 0.602  (0.591-  0.612) | 2.25  (2.04-  2.45) | 0.93  (0.92-  0.95) | 11.2 (10.2-  12.3) | 1.35 (1.23-  1.48) | 99.43  (99.43-  99.44) | 94.49 (94.49-  94.50) | 1.90  (1.80-  2.00) | 0.90  (0.89-  0.91) | 19.0 (18.0-  20.0) | 1.15 (1.09-  1.21) | 99.45  (99.45-  99.46) | 89.57 (89.56-  89.59) |
|  | E | 0.633  (0.616-  0.650) | 3.29  (2.89-  3.68) | 0.88  (0.86-  0.90) | 16.5 (14.6-  18.5) | 1.80 (1.59-  2.02) | 99.51  (99.50-  99.52) | 94.54 (94.52-  94.56) | 2.44  (2.20-  2.68) | 0.84  (0.81-  0.87) | 24.4 (21.9-  26.8) | 1.35 (1.21-  1.48) | 99.53  (99.52-  99.55) | 89.64 (89.62-  89.65) |
|  | F | 0.799  (0.789-  0.808) | 6.02  (5.51-  6.52) | 0.74  (0.71-  0.76) | 30.1 (27.6-  32.5) | 3.26 (2.99-  3.52) | 99.59  (99.58-  99.60) | 94.64 (94.60-  94.68) | 4.65  (4.40-  4.89) | 0.60  (0.57-  0.62) | 46.4 (44.0-  48.9) | 2.53 (2.40-  2.66) | 99.67  (99.65-  99.68) | 89.76 (89.74-  89.78) |
|  | A | 0.819  (0.813-  0.825) | 8.16  (7.79-  8.52) | 0.62  (0.61-  0.64) | 40.7 (39.2-  42.2) | 4.75 (4.54-  4.95) | 99.62  (99.61-  99.63) | 94.67 (94.56-  94.79) | 5.68  (5.45-  5.91) | 0.51  (0.49-  0.53) | 54.1 (52.4-  55.8) | 3.35 (3.22-  3.49) | 99.69  (99.68-  99.70) | 90.24 (90.04-  90.45) |
|  | B | 0.808  (0.798-  0.818) | 8.18  (7.85-  8.52) | 0.63  (0.61-  0.64) | 40.6 (39.5-  41.7) | 4.38 (4.21-  4.55) | 99.65  (99.65-  99.66) | 94.73 (94.62-  94.83) | 5.01  (4.86-  5.16) | 0.51  (0.49-  0.53) | 54.7 (52.7-  56.8) | 2.73 (2.65-  2.81) | 99.72  (99.70-  99.73) | 88.88 (88.73-  89.03) |
| Decision | C | 0.776  (0.752-  0.801) | 6.98  (6.32-  7.65) | 0.68  (0.63-  0.72) | 35.8 (31.3-  40.4) | 3.59 (3.26-  3.92) | 99.64  (99.62-  99.67) | 94.58 (94.31-  94.84) | 5.19  (4.53-  5.86) | 0.63  (0.59-  0.67) | 42.3 (38.3-  46.4) | 2.69 (2.36-  3.03) | 99.67  (99.64-  99.69) | 91.40 (90.48-  92.31) |
| Tree | D | 0.589  (0.579-  0.599) | 2.07  (1.94-  2.20) | 0.95  (0.94-  0.95) | 10.2 (9.5-  10.9) | 1.25 (1.17-  1.33) | 99.43  (99.42-  99.43) | 94.54 (94.33-  94.76) | 1.78  (1.68-  1.88) | 0.91  (0.90-  0.93) | 17.7 (16.6-  18.9) | 1.08 (1.02-  1.13) | 99.45  (99.44-  99.45) | 89.60 (89.39-  89.80) |
|  | E | 0.599  (0.585-  0.613) | 3.16  (2.70-  3.62) | 0.89  (0.87-  0.91) | 15.2 (13.5-  16.9) | 1.73 (1.49-  1.98) | 99.50  (99.49-  99.51) | 94.68 (94.47-  94.89) | 2.33  (2.09-  2.57) | 0.86  (0.83-  0.88) | 23.0 (20.5-  25.4) | 1.29 (1.15-  1.42) | 99.52  (99.51-  99.54) | 89.67 (88.73-  90.61) |
|  | F | 0.779  (0.772-  0.786) | 5.94  (5.47-  6.41) | 0.74  (0.71-  0.76) | 30.1 (27.6-  32.6) | 3.22 (2.98-  3.46) | 99.59  (99.58-  99.60) | 94.58 (94.48-  94.68) | 5.71  (5.13-  6.28) | 0.73  (0.70-  0.75) | 31.2 (29.0-  33.4) | 3.09 (2.79-  3.39) | 99.59  (99.58-  99.61) | 94.13 (93.87-  94.40) |
| Random Forest | A | 0.831  (0.825-  0.836) | 8.12  (7.78-  8.45) | 0.63  (0.61-  0.64) | 40.6 (38.9-  42.3) | 4.73 (4.54-  4.91) | 99.62  (99.61-  99.63) | 94.67 (94.66-  94.68) | 5.55  (5.37-  5.73) | 0.50  (0.47-  0.52) | 55.5 (53.7-  57.3) | 3.28 (3.18-  3.38) | 99.70  (99.69-  99.71) | 89.79 (89.78-  89.80) |

|  | B | 0.836  (0.828-  0.844) | 8.22  (7.75-  8.70) | 0.62  (0.60-  0.65) | 41.1 (38.7-  43.4) | 4.40 (4.16-  4.64) | 99.65  (99.64-  99.67) | 94.71 (94.68-  94.73) | 5.66  (5.44-  5.88) | 0.48  (0.46-  0.51) | 56.4 (54.1-  58.7) | 3.07 (2.96-  3.19) | 99.73  (99.72-  99.74) | 89.85 (89.81-  89.89) |
| --- | --- | --- | --- | --- | --- | --- | --- | --- | --- | --- | --- | --- | --- | --- |
|  | C | 0.788  (0.768-  0.807) | 7.29  (6.57-  8.00) | 0.67  (0.63-  0.71) | 36.4 (32.8-  39.9) | 3.74 (3.39-  4.08) | 99.64  (99.62-  99.66) | 94.69 (94.66-  94.73) | 4.91  (4.49-  5.33) | 0.57  (0.52-  0.61) | 49.1 (44.9-  53.4) | 2.55 (2.34-  2.76) | 99.70  (99.67-  99.73) | 89.78 (89.76-  89.81) |
|  | D | 0.594  (0.583-  0.604) | 2.09  (1.92-  2.25) | 0.94  (0.93-  0.95) | 10.4 (9.6-  11.3) | 1.26 (1.16-  1.36) | 99.43  (99.42-  99.43) | 94.48 (94.47-  94.49) | 1.75  (1.65-  1.84) | 0.92  (0.91-  0.93) | 17.5 (16.5-  18.4) | 1.06 (1.00-  1.11) | 99.44  (99.44-  99.45) | 89.57 (89.55-  89.60) |
|  | E | 0.633  (0.620-  0.647) | 2.87  (2.62-  3.11) | 0.90  (0.89-  0.91) | 14.4 (13.1-  15.6) | 1.58 (1.45-  1.71) | 99.50  (99.49-  99.51) | 94.54 (94.53-  94.55) | 2.37  (2.18-  2.57) | 0.85  (0.83-  0.87) | 23.7 (21.8-  25.6) | 1.31 (1.20-  1.42) | 99.53  (99.52-  99.54) | 89.64 (89.62-  89.67) |
|  | F | 0.801  (0.792-  0.809) | 5.96  (5.50-  6.43) | 0.74  (0.71-  0.76) | 29.8 (27.5-  32.1) | 3.23 (2.99-  3.47) | 99.59  (99.57-  99.60) | 94.64 (94.61-  94.66) | 4.66  (4.47-  4.86) | 0.59  (0.57-  0.61) | 46.7 (44.7-  48.6) | 2.54 (2.44-  2.65) | 99.67  (99.66-  99.68) | 89.76 (89.73-  89.78) |
| XGBoost | A | 0.840  (0.834-  0.846) | 8.93  (8.65-  9.22) | 0.58  (0.57-  0.60) | 44.6 (43.2-  46.1) | 5.18 (5.02-  5.33) | 99.65  (99.64-  99.65) | 94.70 (94.69-  94.70) | 5.81  (5.62-  6.00) | 0.47  (0.45-  0.49) | 58.1 (56.2-  59.9) | 3.43 (3.32-  3.54) | 99.72  (99.70-  99.73) | 89.81 (89.80-  89.82) |
|  | B | 0.842  (0.833-  0.850) | 9.03  (8.67-  9.39) | 0.58  (0.56-  0.60) | 45.3 (43.4-  47.1) | 4.81 (4.63-  4.99) | 99.68  (99.67-  99.69) | 94.71 (94.69-  94.73) | 5.86  (5.54-  6.18) | 0.46  (0.42-  0.49) | 58.7 (55.5-  61.8) | 3.18 (3.01-  3.34) | 99.74  (99.72-  99.76) | 89.82 (89.78-  89.85) |
|  | C | 0.804  (0.785-  0.823) | 7.54  (6.68-  8.40) | 0.66  (0.61-  0.70) | 37.6 (33.6-  41.6) | 3.86 (3.44-  4.28) | 99.65  (99.63-  99.67) | 94.69 (94.63-  94.75) | 5.12  (4.81-  5.44) | 0.54  (0.51-  0.58) | 51.2 (48.0-  54.4) | 2.66 (2.50-  2.82) | 99.71  (99.69-  99.73) | 89.81 (89.77-  89.84) |
|  | D | 0.596  (0.586-  0.606) | 2.18  (2.00-  2.37) | 0.94  (0.93-  0.95) | 10.9 (10.0-  11.9) | 1.32 (1.21-  1.43) | 99.43  (99.42-  99.44) | 94.49 (94.48-  94.50) | 1.85  (1.73-  1.96) | 0.91  (0.89-  0.92) | 18.5 (17.3-  19.6) | 1.12 (1.05-  1.19) | 99.45  (99.44-  99.46) | 89.57 (89.56-  89.58) |
|  | E | 0.628  (0.613-  0.643) | 3.31  (2.90-  3.72) | 0.88  (0.86-  0.90) | 16.6 (14.5-  18.7) | 1.82 (1.60-  2.04) | 99.51  (99.50-  99.52) | 94.55 (94.53-  94.56) | 2.47  (2.24-  2.71) | 0.84  (0.81-  0.86) | 24.7 (22.4-  27.0) | 1.36 (1.24-  1.49) | 99.53  (99.52-  99.55) | 89.64 (89.63-  89.66) |
|  | F | 0.805  (0.795-  0.815) | 6.56  (5.99-  7.13) | 0.71  (0.68-  0.74) | 32.8 (29.9-  35.7) | 3.54 (3.24-  3.83) | 99.61  (99.59-  99.62) | 94.66 (94.64-  94.67) | 4.84  (4.64-  5.04) | 0.57  (0.55-  0.60) | 48.4 (46.4-  50.5) | 2.64 (2.53-  2.74) | 99.68  (99.67-  99.69) | 89.76 (89.75-  89.77) |
| Multi-layer Perceptron | A | 0.836  (0.830-  0.841) | 8.57  (8.23-  8.91) | 0.60  (0.58-  0.62) | 42.8 (41.1-  44.5) | 4.98 (4.79-  5.16) | 99.63  (99.62-  99.64) | 94.69 (94.68-  94.70) | 5.65  (5.50-  5.81) | 0.48  (0.47-  0.50) | 56.5 (55.0-  58.1) | 3.34 (3.25-  3.43) | 99.71  (99.70-  99.72) | 89.80 (89.79-  89.81) |
|  | B | 0.840  (0.831-  0.848) | 8.69  (8.14-  9.25) | 0.60  (0.57-  0.62) | 43.5 (40.8-  46.2) | 4.64 (4.36-  4.92) | 99.67  (99.65-  99.68) | 94.71 (94.68-  94.74) | 5.73  (5.46-  6.00) | 0.48  (0.45-  0.51) | 57.2 (54.5-  59.8) | 3.11 (2.96-  3.25) | 99.73  (99.72-  99.75) | 89.83 (89.80-  89.86) |
|  | C | 0.801  (0.782-  0.821) | 7.38  (6.62-  8.14) | 0.67  (0.63-  0.71) | 36.7 (32.9-  40.4) | 3.78 (3.41-  4.15) | 99.65  (99.63-  99.67) | 94.72 (94.68-  94.77) | 5.12  (4.77-  5.46) | 0.55  (0.51-  0.58) | 50.9 (47.7-  54.2) | 2.65 (2.48-  2.83) | 99.71  (99.69-  99.73) | 89.84 (89.79-  89.88) |
|  | D | 0.595  (0.586-  0.605) | 2.15  (1.98-  2.33) | 0.94  (0.93-  0.95) | 10.8 (9.9-  11.6) | 1.30 (1.19-  1.40) | 99.43  (99.42-  99.44) | 94.49 (94.48-  94.50) | 1.84  (1.73-  1.96) | 0.91  (0.89-  0.92) | 18.4 (17.3-  19.6) | 1.11 (1.05-  1.18) | 99.45  (99.44-  99.46) | 89.56 (89.56-  89.57) |
|  | E | 0.634  (0.618-  0.650) | 3.24  (2.89-  3.58) | 0.88  (0.86-  0.90) | 16.2 (14.4-  17.9) | 1.78 (1.59-  1.97) | 99.51  (99.50-  99.52) | 94.57 (94.55-  94.59) | 2.41  (2.16-  2.67) | 0.84  (0.81-  0.87) | 24.1 (21.5-  26.7) | 1.33 (1.19-  1.47) | 99.53  (99.52-  99.55) | 89.64 (89.62-  89.67) |
|  | F | 0.802  (0.792-  0.812) | 6.43  (5.92-  6.94) | 0.71  (0.69-  0.74) | 32.1 (29.6-  34.7) | 3.47 (3.21-  3.73) | 99.60  (99.59-  99.62) | 94.65 (94.64-  94.67) | 4.81  (4.55-  5.08) | 0.58  (0.55-  0.61) | 48.1 (45.5-  50.8) | 2.62 (2.48-  2.76) | 99.68  (99.66-  99.70) | 89.77 (89.75-  89.78) |

This table is equivalent to Table 1 with the inclusion of 95% confidence intervals

Model A – Socio-demographics, chronic conditions, current pregnancy complications and characteristics Model B – Predictors from Model A, plus previous pregnancy history

Model C – Predictors from Model A, plus grandmother’s pregnancy history, parental birth outcomes Model D – Predictors known at the booking appointment

Model E – Predictors from Model D, plus previous pregnancy history

Model F – Predictors from Model E, plus current pregnancy complications and characteristics

Abbreviations: AUC – Area under the receiving-operator characteristic curve; +LR – Positive likelihood ratio; -LR – Negative likelihood ratio; FPR – alpha (type I error) = 1-specificity; Sensitivity – detection rate, TPR; TP – True Positives; FP

– False Positives; TN – True Negatives; FN – False Negatives; PPV – Positive predictive value = TP/(TP+FP); NPV - Negative predictive value = TN/(FN+TN); CI – Confidence Interval.
